# Supplementary material for: Sensing intracellular calcium ions using a manganese-based MRI contrast agent
Source: Nat Commun. 2019 Feb 22;10:897. doi: 10.1038/s41467-019-08558-7 (PMC6385297; doi:10.1038/s41467-019-08558-7)
Supplement: Supplementary file 1 — Supplementary Information [file 41467_2019_8558_MOESM1_ESM.pdf]

Supplementary Information

Sensing intracellular calcium ions using a manganese-based MRI contrast agent

A Barandov, B B. Bartelle et al.

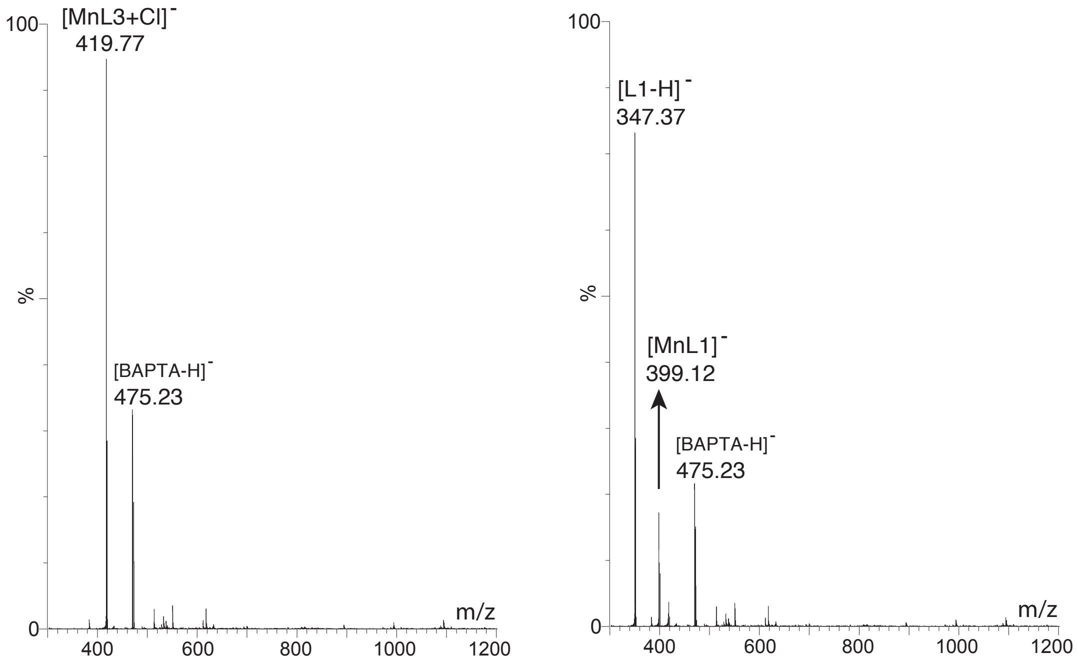

Supplementary Figure 1. MS-spectra of complexes MnL3 (left) and MnL1 (right) in the presence of BAPTA (1.1 equiv). The peak at m/z = 347.37 associating with the free ligand **L1** implies lower stability of Mn**L1** complexes in the presence of BAPTA.

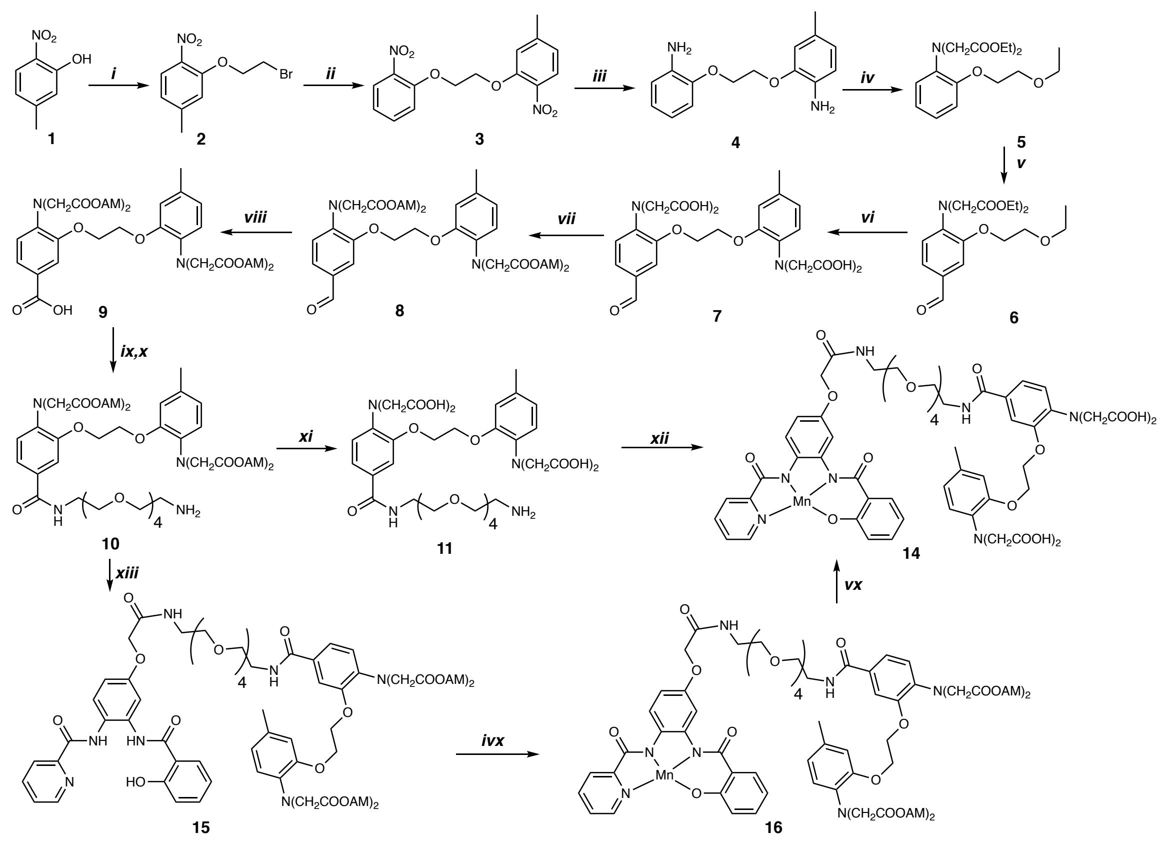

Supplementary Figure 2. Synthesis of bifunctional calcium specific chelators. (*i*) 1,2-dibromoethane, K_2CO_3; (*ii*) 5-methyl-2-nitrophenol, K_2CO_3; (*iii*) Pd/C, H_2; (*iv*) $BrCH_2COOEt$, DIEA, NaI; (*v*) $POCl_3$, DMF, Py; (*vi*) KOH, H_2O; (*vii*) CH_3COOCH_2Br, DIEA; (*viii*) 2-methyl-2-butene, NaH_2PO_4, $NaClO_2$; (*ix*) $BocNHPEG_4NH_2$, PyBop, DIEA; (*x*) TFA, DCM; (*xi*) NaOH, H_2O.

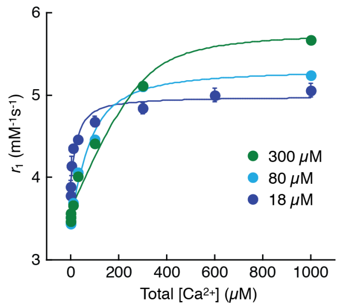

Supplementary Figure 3. Concentration dependence of ManICS1 calcium binding behavior. ManICS1 was formulated at varying concentrations and titrated with calcium concentrations ranging from 0 to 1 mM (total [Ca^{2+}] shown). K_d values were determined by curve fitting to a ligand-depleting bimolecular binding model. Values obtained for ManICS1 concentrations of 18, 80, and 300 μM were 18 ± 12, 40 ± 14, and 33 ± 33 μM, respectively. Although these values were all within error of one another (*t*-test $p \geq 0.26$), weak concentration dependence of ManICS1 calcium sensitivity is indicated by small differences in the relaxivity ranges associated with titration at each probe concentration. Error bars reflect standard deviations from three technical replicates. Note that because ManICS1 buffers calcium, the free calcium concentration in these titrations is lower than the total calcium concentrations noted on the axis, especially for higher concentrations of contrast agent. At low [Ca^{2+}], the apparent ManICS1 concentration dependence of r_1 results primarily from the fact that partial saturation of the sensor is lower for high sensor concentrations.

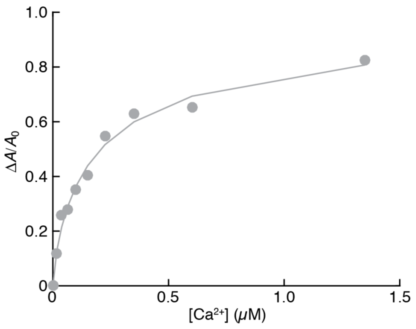

Supplementary Figure 4. Apparent K_d of manganese-free ManICS1. The calcium affinity of manganese free ManICS1 (10 μM) was determined by titration with a spectroscopic readout of absorbance at 230 nm. Buffered calcium concentrations ranging from 0 to 1.5 μM were used (free [Ca^{2+}] shown), and data were fit to a nondepleting bimolecular binding model. The apparent K_d of the compound was determined to be 200 ± 30 nM, similar to the K_d of BAPTA itself.

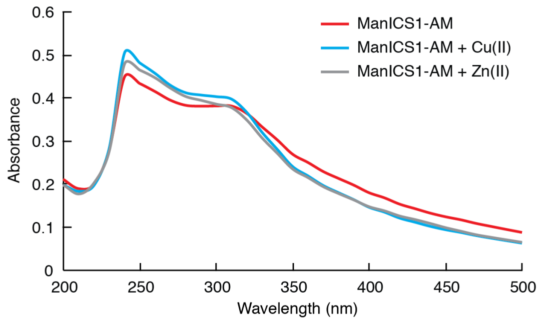

Supplementary Fig. 5. Stability of ManICS1-AM against transmetallation. ManICS1-AM (40 µM, red) in Bis-Tris (50% EtOH, pH 7.4, 50 mM) was treated with solutions of $CuCl_2$ (50 µM) and $ZnCl_2$ (50 µM) in Bis-Tris (50% EtOH, pH 7.4, 50 mM) and stirred at room temperature for 5 h before recording of the optical absorbance spectra shown here.

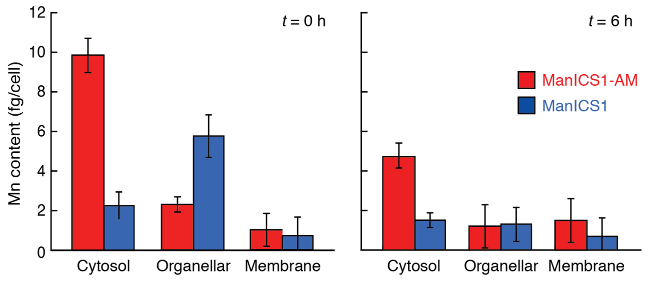

Supplementary Figure 6. Elemental analysis of manganese distribution in cells incubated with ManICS1 and ManICS1-AM. HEK293 cells incubated with 10 µM ManICS1-AM (red) or ManICS1 (blue) and then washed. Cell fractionation was performed either immediately after washing (left) or following a further 6 hour incubation and additional wash (right). The distribution of manganese at the two time points was determined by quantifying the manganese levels in known quantities of cells using inductively coupled plasma optical emission spectroscopy (ICP-OES). Relative localization of the two probes is consistent with the hypothesis that ManICS1-AM is internalized by cells and retained in cytosols, while ManICS1 is taken up by endocytosis and not effectively retained.

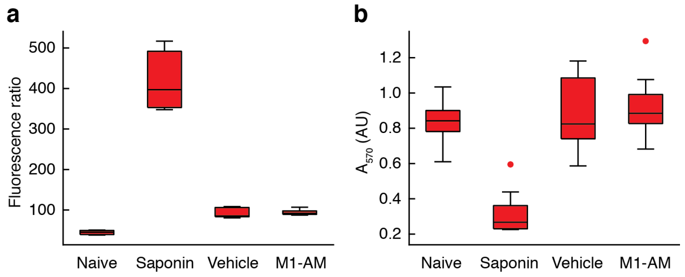

Supplementary Figure 7. ManICS1-AM incubation is nontoxic. (a) In an assay for membrane disruption and toxicity using Ethidium Homodimer III cells incubated in 100 μM ManICS1-AM (M1-AM) do not show a significantly increased fluorescence ratio (530/620 nm) compared with vehicle-treated controls (p = 0.065, n = 6), consistent with the absence of acute toxicity. In contrast, saponin treatment is acutely toxic, resulting in substantially more ethidium uptake. **(b)** In an MTT assay for cellular metabolic activity via NAD(P)H dependent oxidoreductases, there is no loss of activity in cells treated with ManICS1-AM or vehicle, with respect to naive controls ($p \geq 0.3$, n = 8). Again, saponin treatment reduces cell health, resulting in a lower absorbance signature. All boxes denote median readouts (center lines), first and third quartile boundaries (box limits), and full data ranges (whiskers), with outliers denoted by red dots.

SUPPLEMENTARY METHODS

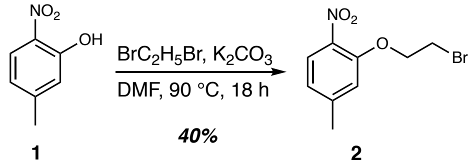

2-(2-bromoethoxy)-4-methyl-1-nitrobenzene, (**2**). 5-methyl-2-nitrophenol (9 g, 59 mmol) and K_2CO_3 (4.9 g, 35 mmol) were suspended in anhydrous DMF (24 mL) and stirred at 90 °C for 20 min. 1,2-dibromoethane (66 g, 351 mmol) was added and the reaction mixture was stirred at 90 °C for 18 h. After removal of all volatiles the product was purified by column chromatography (ethyl acetate : hexane (1:4), silica). Yield: 6 g (40%). ^{1}H-NMR (400 MHz, $CDCl_3$): δ = 7.77 (d, 1H, J_{HH} = 8.4 Hz), 6.8 (m, 2H), 4.39 (t, 2H, J_{HH} = 6.4 Hz), 3.67 (t, 2H, J_{HH} = 6.4 Hz), 2.41 (s, 3H). ^{13}C-NMR (101 MHz, CDCl3): δ = 151.70, 146.00, 138.06, 126.02, 122.21, 116.04, 77.48, 77.16, 76.84, 69.71, 28.21, 22.01.

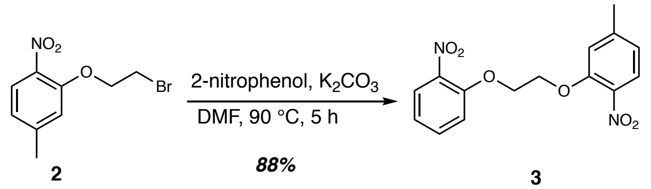

4-methyl-1-nitro-2-(2-(2-nitrophenoxy)ethoxy)benzene, (**3**). Compound **2** (2.6 g, 10 mmol), 2-nitrophenol (1.4 g, 10 mmol) and K_2CO_3 (0.83 g, 6 mmol) were suspended in anhydrous DMF (10 mL) and stirred at 90 °C for 5 h. After cooling the reaction mixture to room temperature distilled water (100 mL) was added slowly and the resulting solid was filtered and washed with water and diethyl ether and dried in vacuum to afford **3** as white solid. Yield: 2.8 g (88%). ^{1}H-NMR (400 MHz, DMSO_d6): δ = 6.86 (m, 1H), 6.72-6.63 (m, 3H), 6.55-6.49 (m, 3H), 4.26 (s, 4H), 2.16 (s, 3H). ^{13}C-NMR (101 MHz, DMSO_d6): δ = 145.40, 145.35, 138.17, 135.49, 124.97, 121.63, 121.46, 116.19, 114.21, 114.14, 113.62, 112.70, 67.26, 67.17, 40.15, 39.94, 39.73, 39.31, 39.10, 38.89, 20.53.

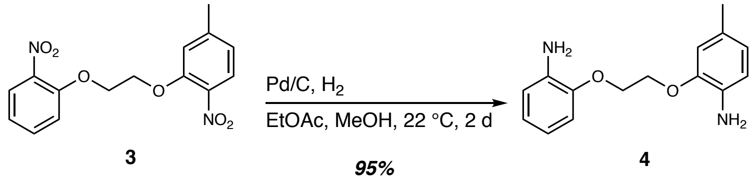

2-(2-(2-aminophenoxy)ethoxy)-4-methylaniline, (**4**). Compound **3** (2 g, 6.3 mmol) and Pd/C (0.4 g, 10 wt%) were suspended in a mixture of EtOAc/MeOH (8:2, 200 mL) and the mixture was stirred under hydrogen atmosphere at room temperature for 2 days. The catalyst was filtered off over sand and after removal of all volatiles the residue was washed with MeOH and dried in vacuum. Yield: 1.5 g (95%). MS (HRESI): m/z: 259.3312 [M+H]+. ^{1}H-NMR (400 MHz, DMSO_d6): δ = 6.85 (m, 1H), 6.71-6.63 (m, 3H), 6.55-6.49 (m, 3H), 4.67 (s, 2H),4.46 (s, 2H), 4.26 (s, 4H), 2.16 (s, 3H). ^{13}C-NMR (100 MHz, Code: AB 6, DMSO_d6): δ = 145.39, 145.35, 138.17, 135.49, 124.96, 121.62, 121.45, 116.19, 114.20, 114.14, 113.62, 112.70, 67.26, 67.17, 20.53.

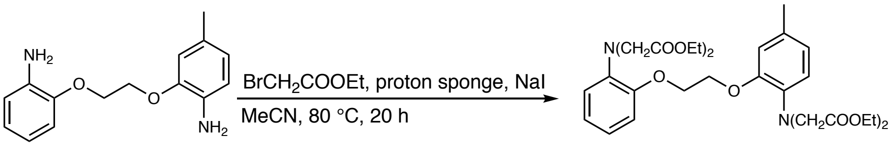

4 *70%* 5

tetra ethyl [1-(o-aminophenoxy)2-(4-methyl-o-aminophenoxy)ethane]-N,N,N',N'-tetraacetate, (**5**). Compound **4** (2.6 g, 10 mmol), ethyl bromoacetate (8.35 g, 50 mml) and proton sponge (10.7 g, 50 mmol) were dissolved in anhydrous MeCN (100 mL) and NaI (8 g, 53 mmol) was added. The resulting mixture was stirred under reflux for 20 h. After cooling to room temperature, the mixture was diluted with DCM (300 mL) and washed with saturated aqueous solutions of NaH_2PO_4, $NaHCO_3$ and NaCl successively. The organic phase was dried over MgSO4 and after removal of all volatiles the residue was suspended in MeOH (20 mL) and cooled to - 20 °C to afford **5** as pale yellow microcrystalline solid. Yield: 4.2 g (70%). MS (HRESI): m/z: 603.2908 $[M+H]^+$. ^{1}H-NMR (400 MHz, $CDCl_3$): δ = 6.91-6.80 (m, 4H), 6.76-6.74 (m, 1H), 6.66-6.64 (m, 2H), 4.26 (br, 4H), 4.14 (s, 4H), 4.12 (s, 4H), 4.03 (q, 8H), 2.24 (s, 3H), 1.13 (td, 12H). ^{13}C-NMR (101 MHz, $CDCl_3$): δ = 171.57, 171.52, 150.40, 150.33, 139.40, 136.73, 132.22, 122.26, 121.77, 121.52, 119.35, 119.11, 114.25, 113.39, 67.20, 67.09, 60.77, 60.71, 53.69, 53.88, 21.00, 14.10.

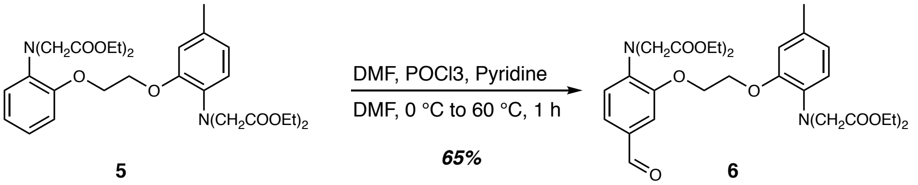

5 *65%* 6

diethyl 2,2'-((2-(2-(2-(bis(2-ethoxy-2-oxoethyl)amino)-5-formylphenoxy)ethoxy)-4-methylphen yl)azanediyl)diacetate, (**6**). Compound **5** (2 g, 3.3 mmol) and pyridine (0.4 mL) were dissolved in anhydrous DMF (20 mL) and cooled in an ice bath under argon atmosphere. $POCl_3$ (3 mL) was added dropwise and the mixture was warmed to 60 °C and stirred at this temperature for 1 h (TLC showed completion of the reaction). The reaction solution was diluted with DCM (40 mL) and added to NaOH solution mixed with crushed ice. The organic phase was diluted with DCM (200 mL) and washed with $NaHCO_3$ and NaCl solutions and after workup and removal of all volatiles the residue was triturated with MeOH and cooled to -20 °C. Overnight yellow crystalline solid of **6** was isolated which was filtered and washed with MeOH and dried in vacuum. Yield: 1.4 g (65%). MS (HRESI): m/z: 631.29011 [M+H]+. 1H-NMR (400 MHz, CDCl3): δ = 9.77 (s, 1H), 7.37-7.36 (m, 2H), 6.76-6.65 (m, 2H), 4.031-4.25 (m, 4H), 4.21 (s, 4H), 4.10 (s, 4H), 4.08-4.00 (m, 8H), 2.24 (s, 3H), 1.15-1.13 (m, 12). 13C-NMR (101 MHz, CDCl3): 190.56, 171.49, 170.88, 150.23, 149.75, 145.28, 132.26, 129.99, 126.60, 122.09, 67.52, 66.89, 61.26, 60.77, 53.83, 53.69, 21.02, 14.14, 14.09.

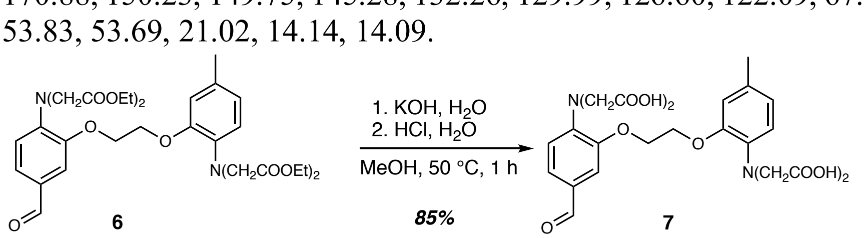

6 *85%* 7

2,2'-((2-(2-(2-(bis(carboxymethyl)amino)-5-formylphenoxy)ethoxy)-4-methylphenyl)aza- nediyl)diacetic acid, (**7**). Compound **6** (700 mg, 1.1 mmol) was

dissolved in warm (50 °C) methanol (5 mL) and KOH (370 mg, 6.6 mmol) in deionized water (20 mL) was added. After stirring the reaction solution at 50 °C for 2 h the solvent volume was reduced to 10 mL in vacuum and the solution was neutralized by adding HCl (1N). The resulting precipitate was dissolved in THF, dried over $MgSO_4$ and after removing of all volatile in vacuum yellow solid was obtained. Yield: 490 mg (85%). MS (HRESI): m/z: 517.1455 $[M-H]^-$. ^{1}H-NMR (400 MHz, MeOD-*d*4): δ = 9.77 (s, 1H), 7.65 (m, 2H), 7.51-7.49 (m, 1H), 7.12-6.71 (m, 5H), 4.44-3.84 (m, 12H), 2.29 (s, 3H). ^{13}C-NMR (101 MHz, MeOD-*d*4): δ = 129.79,j 175.86, 175.63, 175.54, 175.39, 152.00, 150.81, 146.65, 144.79, 137.50, 127.32, 124.91, 122.66, 120.81, 119.82, 119.04, 117.63, 67.51, 56.22, 56.10, 21.17.

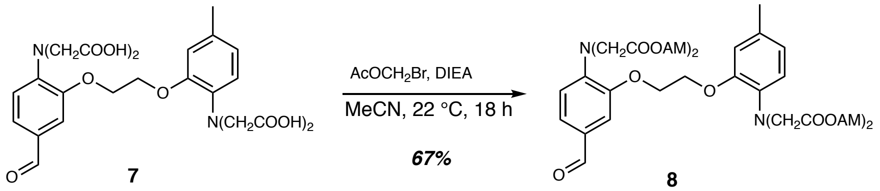

bis(acetoxymethyl) 2,2'-((2-(2-(2-(bis(2-(acetoxymethoxy)-2-oxoethyl)amino)-5-formylphenoxy) ethoxy)-4-methylphenyl)azanediyl)diacetate, (**8**). Compound **7** dissolved in anhydrous acetonitrile (10 mL) and bromomethyl acetate (1 g, 6 mmol) and diisopropylethylamine (0.9 g, 7 mmol) were added. After stirring the solution for 18 h al volatile were removed in vacuum and the residue was purified by column chromatography (silica, Ethyl acetate: hexane, 1:1) to afford **8** as colorless oil. Yield: 0.6 g (67%). MS (HRESI): m/z: 807.7213 $[M+H]^+$. 1H-NMR (400 MHz, $CDCl_3$): δ = 9.79 (s, 1H), 7.40 (m, 2H), 6.81-6.77 (m, 2H), 6.71-6.67 (m, 2H), 5.61 (s, 4H), 5.60 (s, 4H), 4.35-4.33 (m, 2H), 4.29-4.27 (m, 2H), 4.27 (s, 4H), 4.14 (s, 4H), 2.26 (s, 3H), 2.06 (s, 6H), 2.05 (s, 6H). ^{13}C-NMR (101 MHz, $CDCl_3$): δ = 190.61, 170.20, 169.65, 169.59, 169.52, 150.57, 149.92, 144.57, 136.35, 133.18, 130.59, 126.58, 122.32, 120.43, 117.34, 114.82, 111.34, 79.59, 79.27, 77.48, 77.16, 76.84, 67.49, 66.95, 53.58, 53.48, 21.14, 21.06, 20.74.

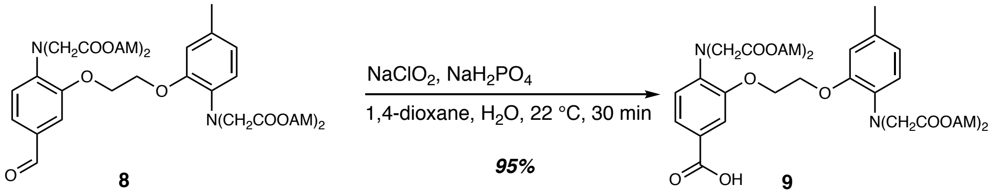

4-(bis(2-(acetoxymethoxy)-2-oxoethyl)amino)-3-(2-(2-(bis(2-(acetoxymethoxy)-2-oxoethyl) amino)-5-methylphenoxy)ethoxy)benzoic acid, (**9**). Compound **8** (400 mg) and 2-methylbut-2-ene (100 µL) were dissolved in 1,4-dioxane (5 mL) and aqueous solutions of NaH_2PO_4 (180 mg, 1 mL) and $NaClO_2$ (110 mg, 1 mL) were added successively and the resulting solution stirred at room temperature for 30 min. A second batch of NaH_2PO_4 (180 mg, 1 mL) and $NaClO_2$ (110 mg, 1 mL) were added and after additional 30 min stirring at room temperature the reaction progress was monitored by TLC. The reaction was quenched by adding water (50 mL) and extracted with EtOAc (3 x 50 mL). The organic phases were combined, dried over MgSO4 and after removal of all volatiles in vacuum **9** was obtained as colorless oil and used without further purification. Yield: 390 mg (95%). MS (HRESI): m/z: 821.7203 $[M-H]^-$. ^{1}H-NMR (400 MHz, $CDCl_3$): δ = 7.35-7.33 (m, 1H), 6.49-6.37 (m, 5H), 5.31 (s, 4H), 5.28 (s, 4H), 4.04-3.95 (m, 8H), 3.84 (S, 4H), 1.75 (s, 3H), 1.74 (s, 6H), 1.73 (S, 6H). ^{13}C-NMR (101 MHz, $CDCl_3$): δ =

170.75, 170.62, 170.17, 169.72, 169.59, 169.54, 169.48, 168.82, 167.12, 160.97, 160.28, 153.68, 150.50, 149.13, 148.88, 143.53, 143.38, 141.18, 136.23, 132.99, 130.39, 125.34, 124.56, 122.61, 122.45, 122.11, 121.86, 120.21, 117.31, 114.63, 114.16, 113.71, 79.44, 79.19, 77.48, 77.16, 76.84, 67.03, 53.49, 53.40, 20.94, 20.62.

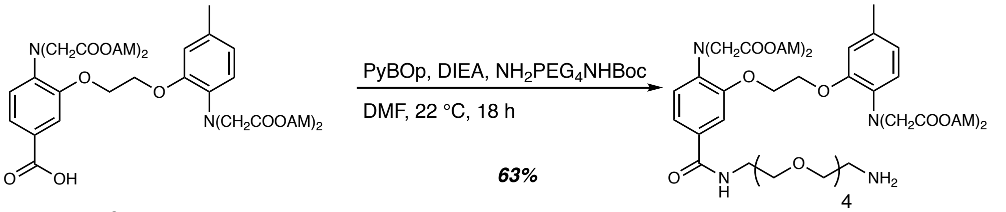

9 **10**

4-(bis(2-(acetoxymethoxy)-2-oxoethyl)amino)-3-(2-(2-(bis(2-(acetoxymethoxy)-2-oxoethyl) amino)-5-methylphenoxy)ethoxy)-*N*-(14-amino-3,6,9,12-tetraoxatetra-decyl)benzamide, (10). Compound **9** (29 mg, 0.035 mmol) and DIEA (6.8 mg, 0.053 mmol, 9 µL) were dissolved in anhydrous DMF (3 mL) and PyBop (19 mg, 0.035 mmol) was added. After stirring the reaction solution at room temperature for 10 min, NH_2PEG_4NHBoc (17 mg, 0.053 mmol) was added and the solution was stirred at ambient temperature for 18 h. All volatiles were removed in vacuum and the resulting residue was purified by column chromatography on silica gel eluting with DCM:EtOAc (2:1). The fractions showing expected mass of tert-butyloxy protected intermediate were pooled and after removal of solvent the resulting residue was dissolved in DCM:TFA (10:1) mixture and stirred at ambient temperature for 3 h. All volatiles were removed in the vacuum to obtain **10** as colorless oil. Yield: 23 mg (63%). MS (HRESI): m/z = 1041.4140 $[M+H]^+$. ^{1}H-NMR (400 MHz, $CDCl_3$): δ = 7.47 (s, 1H), 7.33-7.31 (m, 1H), 6.80-6.68 (m, 4H), 5.62 (s,4H), 5.60 (s,4H), 4.36-4.28 (m, 4H), 4.23 (s, 4H), 4.16 (s,4H), 3.66-3.59 (m, 16H), 3.50 (t, 2H, J_{HH} = 5.2 Hz), 3.28-3.26 (m, 2H), 3.08-3.07 (m, 2H), 2.27 (s, 3H), 2.06 (d, 12H, J_{HH} =4.7 Hz). ^{13}C-NMR (101 MHz, $CDCl_3$): δ = 170.20, 169.86, 169.56, 169.50, 150.52, 149.73, 141.38, 133.04, 128.34, 122.01, 120.23, 117.95, 117.46, 116.64, 114.52, 112.52, 79.33, 79.18, 77.34, 77.23, 77.02, 76.71, 70.43, 67.33, 66.93, 53.40, 53.29, 46.35, 46.30, 39.80, 37.03, 35.57, 28.41, 26.45, 26.37, 20.68.

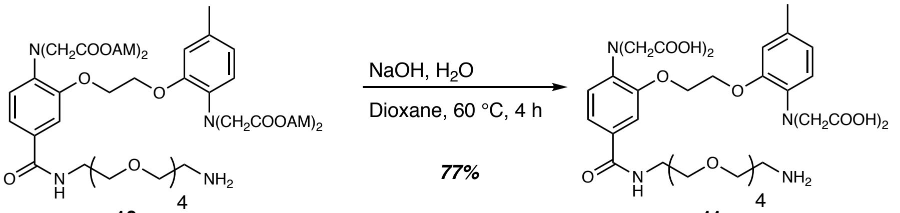

10 **11**

2,2'-((4-((14-amino-3,6,9,12-tetraoxatetradecyl)carbamoyl)-2-(2-(2-(bis(carboxy-methyl)amino)-5-methylphenoxy)ethoxy)phenyl)azanediyl)diacetic acid, (11). Compound **10** (20 mg, 0.02 mmol) was dissolved in 1,4-dioxane (5 mL) and a solution of NaOH (10 mg, 0.25 mmol) in distilled water (5 mL) was added. After stirring the mixture at 60 °C for 30 min, the reaction solution was cooled to room temperature and stirred for additional 4 h. The completion of reaction was confirmed by HPLC. Next, all volatiles were removed in vacuum and the resulting residue was dissolved in H2O and acidified by HCl (1N) solution to pH 2.5. The resulting precipitate was filtered and dried in vacuum to afford **11** as pale yellow solid. Yield: 11 mg (77%). MS (HRESI): m/z = 751.3022 $[M-H]^-$. ^{1}H-NMR (400 MHz, MeOD-d4): δ = 7.51 (s, 1H), 7.41 (d, 1H, J_{HH} = 8 Hz), 6.85-6.69 (m,

5H), 4.39-4.38 (m, 4H), 4.35 (s, 6H), 4.17 (s, 4H), 4.15-4.05 (m, 16H), 3.67 (s, 8H), 3.26 (s, 2H), 313-3.09 (m, 2H), 2.28 (s, 1H). ^{13}C-NMR (101 MHz, MeOD-d4): δ = 175.79, 175.79, 175.14, 137.74, 134.03, 126.75, 125.18, 122.80, 122.15, 120.39, 117.78, 115.59, 115.34, 114.19, 114.10, 71.40, 71.30, 70.96, 70.82, 67.77, 55.80, 55.18, 40.88, 40.62, 21.03.

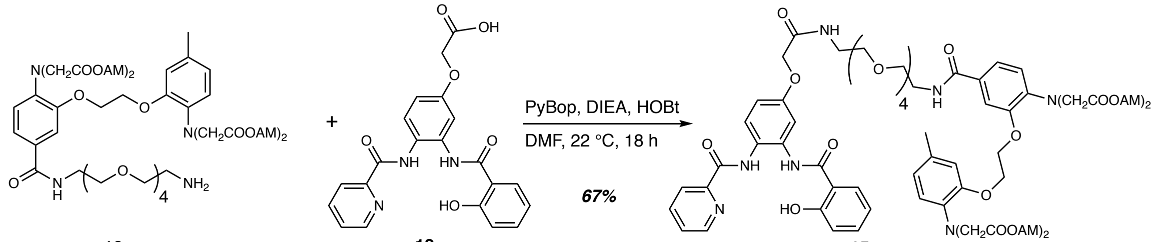

apo-ManICS1-AM, (**15**). Compound **12** (21 mg, 0.05 mmol) and DIEA (13 mg, 0.1 mmol) were dissolved in DMF (3 mL) and PyBop (32 mg, 0.06 mmol) and HOBt (8 mg, 0.06 mmol) were added. The reaction solution stirred for 10 min at ambient temperature and a solution of **10** (64 mg, 0.06 mmol) in DMF (1 mL) was added. After stirring the solution at ambient temperature for 18 h, all volatiles were removed in vacuum and the product was purified by column chromatography on silica gel eluting with DCM:MeOH (9.5:0.5) to afford **15** as pale yellow oil. Yield: 49 mg (67%). MS (HRESI): m/z = 1430.5046 [M+H]$^+$. ^{1}H-NMR (400 MHz, $CDCl_3$): δ = 10.27 (s, 1H), 8.60 (s, 1H), 8.29 (d, 1H, J_{HH} = 8 Hz), 7.94-7.89 (m, 1H), 7.81 (d, 1H, J_{HH} = 8 Hz), 7.52-7.35 (m, 8H), 7.26-6.67 (m, 8H), 5.62 (s, 4H), 5.59 (s, 4H), 4.49 (s, 2H), 4.25 (s, 4H), 4.21 (s, 4H), 4.14 (s,4H), 3.62-3.41 (m, 22H), 2.25 (s, 3H), 2.06 (s, 6H), 2.04 (s, 6H). ^{13}C NMR (101 MHz, CDCl3) δ 170.25, 169.87, 169.60, 169.57, 169.50, 162.05, 160.14, 150.55, 148.72, 148.46, 147.63, 139.34, 139.15, 137.80, 136.62, 135.91, 134.61, 133.23, 126.98, 126.72, 125.84, 124.38, 122.60, 120.31, 118.98, 118.49, 117.98, 112.30, 79.40, 79.24, 77.32, 76.68, 70.12, 53.49, 46.39, 26.41, 26.33, 20.62.

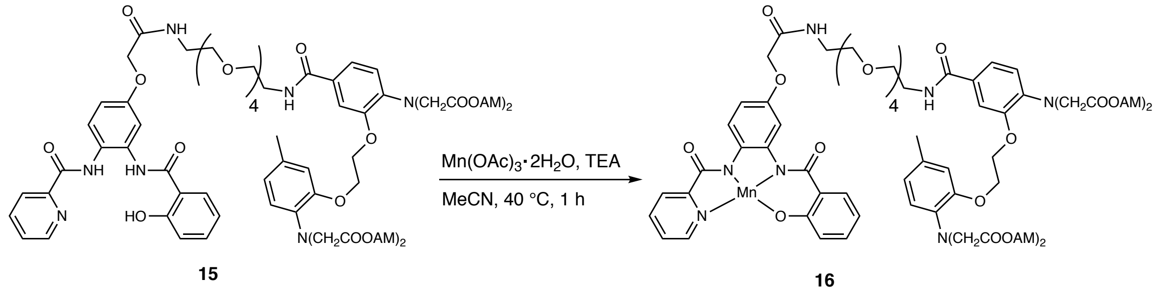

ManICS1-AM, (**16**). Compound **15** (20 mg, 0.014 mmol) was dissolved in MeCN (5 mL) and $Mn(OAc)_3 \bullet 2H_2O$ (4 mg, 0.015 mmol) was added and the reaction mixture was stirred at room temperature for 5 min. Next, triethylamine (4.2 mg, 0.045 mmol, 6 µL) was added and the brown reaction solution was stirred at 40 °C for 1 h. After removal of all volatiles the resulting dark brown solid was suspended in cold (-20 °C) methanol (2 mL) and filtered to afford **16** as dark brown solid. Yield: 16 mg (77%). MS (HRESI): m/z = 1482.4197 (theoretical) [M+H]$^+$, 1482.4176 (experimental) [M+H]$^+$, 1504.4004 [M+Na]$^+$.

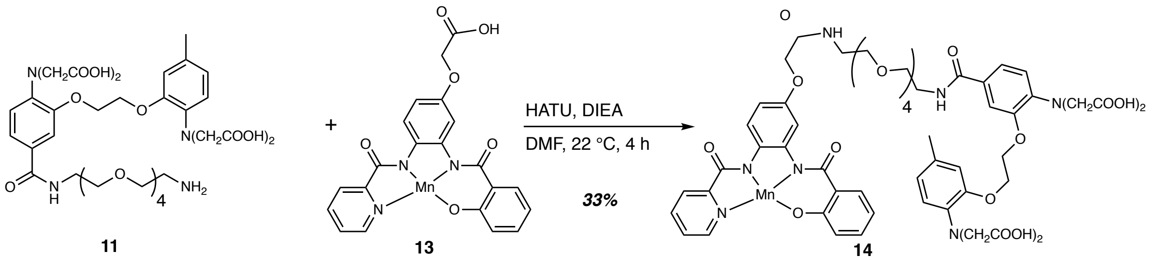

ManICS1, (**14**). *Method A*: Compound **13** (20 mg, 0.04 mmol) was dissolved in anhydrous DMF (3 mL) and DIEA (28 mg, 0.6 mmol, 38 µL) was added. After stirring the reaction solution for 5 min at rom temperature HATU (19 mg, 0.05 mmol) was added and the solution was stirred for additional 30 min. Next, compound **11** (38 mg, 0.05 mmol) was added and the reaction solution was stirred at ambient temperature for 2 h. The reaction was quenched with adding H_2O (10 µM) and purified by high performance liquid chromatography (silica-C18, eluent gradient H_2O:MeCN from 95:5 to 10:90). t_R = 9.1 min. Yield: 16 mg (33%). MS (HRESI): m/z = 1192.3195 (theoretical) $[M-H]^-$, 1192.3202 (experimental) $[M-H]^-$, 1228.2933 $[M+Cl]^-$.

Method B: A solution of **ManICS1-AM** (50 µM, 100 µL) in MOPS buffer (pH 7.9, 25 mM, KCl 100 mM) was added to freshly harvested HEK-293 cell-lysate (900 µL, 100×10^6 cells/mL) and incubated at 37 °C for 5 h. The HPLC trace of the mixture confirmed full hydrolysis of **ManICS1-AM** to its tetracarboxylic acid form **ManICS1**. Yield: 92% (based on HPLC trace).

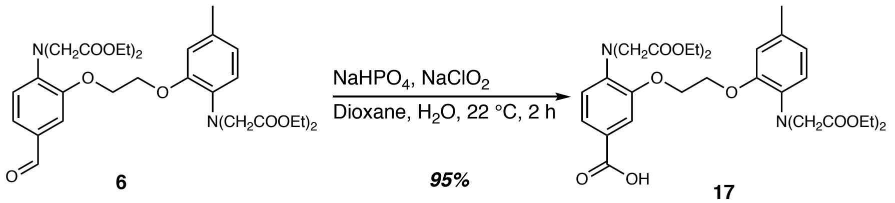

4-(bis(2-ethoxy-2-oxoethyl)amino)-3-(2-(2-(bis(2-ethoxy-2-oxoethyl)amino)-5-methylphenoxy)ethoxy)benzoic acid, (**17**). Compound 6 (60 mg, 0.1 mmol) was dissolved in 1,4-dioxane (5 mL) and 2-methyl-2-nutene (10 µL) was added. NaH_2PO_4 (36 mg, 0.3 mmol) and $NaClO_2$ (25 mg, 0.25 mmol) were dissolved in distilled water separately (5 mL total volume of water) and added successively to the reaction solution. The resulting mixture was stirred vigorously for 2 h and extracted with ethyl acetate (3 x 50 mL). After washing the organic phase with aqueous solution of Na_2SO_3 (100 mM) and drying over $MgSO_4$, all volatiles were removed in vacuum to afford **17** as yellow oil, which was used without further purifications. MS (HRESI): *m/z* = 669.6823 $[M+Na]^+$. ^{1}H NMR (400 MHz, $CDCl_3$): δ = 9.78 (s, 1H), 7.38 (d, 2H, J_{HH} = 7.1 Hz), 7.30 – 7.26 (m, 1H), 6.83 – 6.67 (m, 3H), 5.28 (s, 1H), 4.86 (d, 1H, J_{HH} = 17.4 Hz), 4.55 – 3.80 (m, 20H), 2.34 (s, 3H), 1.20 (dt, 10H, J_{HH}= 15.5, 7.1 Hz), 0.98 (t, 3H J_{HH}= 7.1 Hz). ^{13}C NMR (101 MHz, $CDCl_3$): δ = 190.62, 170.77, 168.38, 162.27, 161.95, 153.74, 149.44, 145.24, 140.96, 130.40, 130.07, 126.89, 126.30, 121.85, 117.06, 113.65, 111.84, 77.48, 77.16, 76.84, 67.18, 66.77, 61.61, 61.35, 61.19, 54.01, 53.55, 48.71, 21.74, 18.06, 14.21, 13.74.

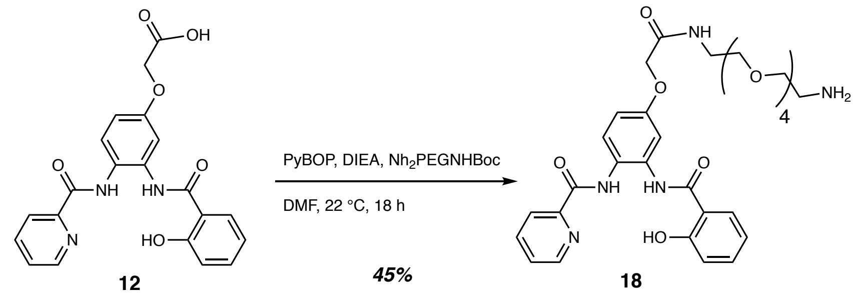

***N*-(4-((17-amino-2-oxo-6,9,12,15-tetraoxa-3-azaheptadecyl)oxy)-2-(2-hydroxy benzamido)phenyl)picolinamide, (18).** Compound **12** (40 mg, 0.1 mmol) was dissolved in anhydrous DMF (5 mL) and DIEA (0.26 mg,0.2 mmol) was added. After stirring at room temperature for 5 min, PyBop (52 mg, 0.1 mmol) and HOBt (13 mg, 0.1 mmol) were added and stirred for 10 min. Next, NH_2PEGNHBoc (50 mg, 0.15 mmol) was added and the reaction stirred at ambient temperature for 18 h. All volatiles were evaporated in vacuum and product was purified by column chromatography (silica gel, DCM:MeOH, 93:7). The isolated intermediate was dissolved in DCM:TFA (1:1) solution and stirred at room temperature for 1 h. All volatiles were remover and the residue was dissolved in H_2O (3 mL) and freeze-dried to afford product as yellow solid. Yield: 28 mg (45%). MS (HRESI): *m/z* = 624.2644 $[M-H]^-$. ^{1}H NMR (400 MHz, $CDCl_3$): δ =10.25 (s, 1H), 8.59-8.58 (d, 1H, J_{HH}= 4 Hz), 8.30-8.28 (m, 1H), 7.91-7.83 (m, 2H), 7.68 (d, 1H, J_{HH}= 8 Hz), 7.50-7.39 (m, 5H), 7.38-7.31 (m, 1H), 7.00-6.84 (m, 2H), 3.63-3.47 (m, 20H), 3.27-3.26 (m, 2H). ^{13}C NMR (101 MHz, $CDCl_3$): δ = 168.69, 168.02, 163.50, 161.89, 155.66, 148.85, 148.49, 137.83, 134.50, 131.84, 128.59, 127.05, 127.00, 126.25, 124.42, 122.62, 119.01, 118.44, 112.54, 70.57, 70.52, 70.40, 70.27, 69.72, 67.83, 54.70, 46.38, 46.33, 38.93, 28.49, 26.45, 26.37.

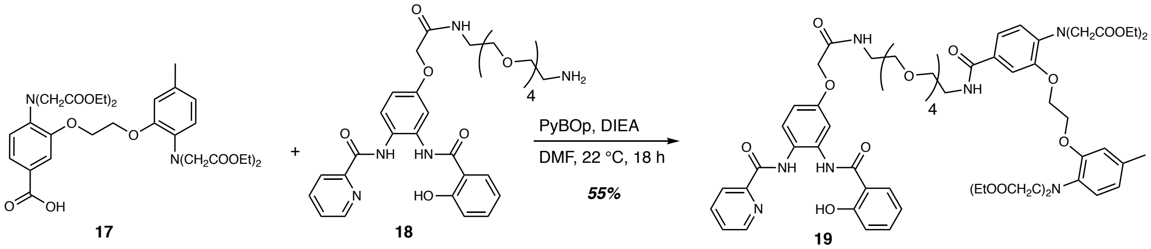

apo-ManICS1-Et, (**19**). Compound **17** (20 mg, 0.031 mmol) and DIEA (5.2 mg, 0.04 mmol) were dissolved in anhydrous DMF (3 mL) and PyBOP (17 mg, 0.032 mmol) was added and the reaction solution was stirred at room temperature for 10 min. Next, **18** (23 mg, 0.031 mmol) was added and the reaction solution was stirred for additional 18 h. After removal of all volatiles in vacuum the residue was purified by column chromatography (silica gel, EtOA:DCM, 1:1) to afford **19** as pale yellow oil. Yield: 21 mg (55%). MS (HRESI): *m/z* = 1254.5433 $[M+H]^+$, 1276.5260 $[M+Na]^+$. ^{1}H NMR (400 MHz, $CDCl_3$): δ = 10.28 (s, 1H), 8.61 (d, 1H, J_{HH} = 8 Hz), 8.30 (d, 1H, J_{HH} = 8 Hz), 7.94-7.80 (m, 5H), 7.52-7.35 (m, 7H), 7.26-6.68 (m, 9H), 4.23-3.56 (m, 20H), 3.55-3.45 (m, 20H), 2.36 (s, 3H), 1.29-1.17 (m, 12H). ^{13}C NMR (101 MHz, $CDCl_3$): δ = 169.32, 168.45, 163.40, 161.19, 160.87, 160.83, 155.36, 148.71, 148.33, 139.06, 137.75, 134.41, 131.48, 128.44, 127.83, 127.12, 126.81, 126.57, 125.96, 125.82, 124.58, 122.42, 119.19, 118.04, 116.25, 115.02, 114.79, 114.59, 112.59, 112.40, 111.64, 77.48, 77.36, 77.16, 76.84, 70.09, 70.00,

69.85, 69.79, 69.76, 69.68, 69.56, 69.42, 67.32, 66.74, 54.22, 47.83, 47.78, 45.37, 42.45, 40.03, 38.80, 26.04, 25.95, 24.25, 18.47, 17.16, 12.22.

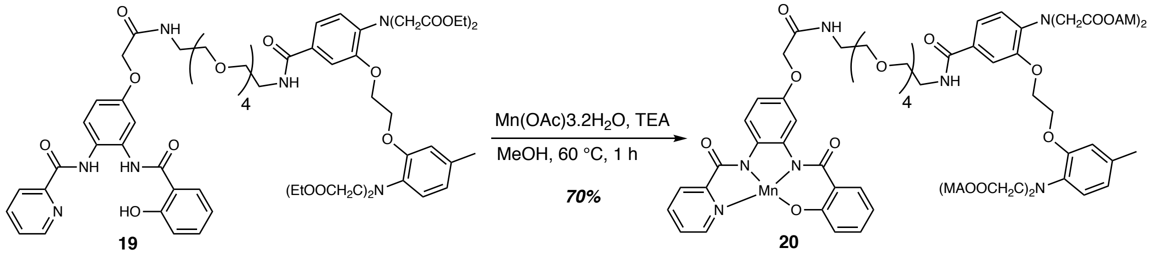

ManICS1-Et, (**20**). Compound **19** (25 mg, 0.02 mmol) was dissolved in MeOH (5 mL) and $Mn(OAc)_3•2H_2O$ (5.5 mg, 0.021 mmol) and stirred at 60 °C for 10 min. Next triethyl amine (6 mg, 0.06 mmol) was added and the reaction solution was stirred at 60 °C for additional 50 min. After cooling to room temperature the precipitated solid was filtered and washed with methanol and dried in vacuum to afford **20** as dark brown solid. HPLC (silica-C18, eluent gradient H_2O:MeCN from 95:5 to 10:90) trace of **20** revealed a single peak at t_R = 18.4 min. Yield: 18 mg (70%). MS (HRESI): m/z = 1306.4603 (theoretical) $[M+H]^+$, 1306.4592 (experimental) $[M+H]^+$, 1328.4421 $[M+Na]^+$.

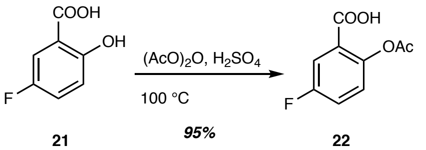

2-acetoxy-5-fluorobenzoic acid, (**22**). 2-hydroxy-5-fluorobenzoic acid (1.5 g, 0.01 mol) was dissolved in acetic anhydride (10 mL) and treated with catalytic amount of H_2SO_4 (0.5 mL). The solution was heated at 100 °C for 20 min and cooled to 0 °C. The solution was treated with cold water (5 °C) and the precipitated solid filtered off and washed with water (4 x 10 mL). After dying the compound in vacuum overnight, **22** was obtained as white solid. Yield: 1.9 g (95%). ^{1}H NMR (400 MHz, $CDCl_3$): δ = 7.82 – 7.79 (m, 1H), 7.33 – 7.30 (m, 1H), 7.14 – 7.10 (m, 1H), 2.35 (s, 3H). ^{13}C NMR (101 MHz, $CDCl_3$): δ = 173.78, 169.83, 168.82, 160.97, 125.7 (d), 124.68, 122.01 (d), 119.08 (d), 20.92. ^{19}F NMR (376 MHz, $CDCl_3$) δ -114.93 (m).

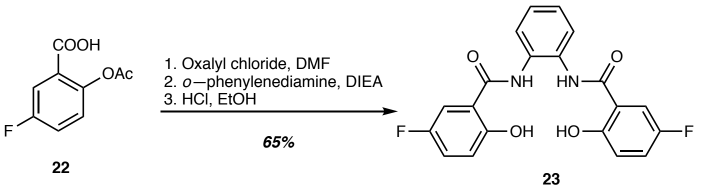

L1F, (**23**). Compound **22** (3 g, 0.015 mol) was dissolved in DCM and oxalyl chloride (2.1 g, 0.016 mol) was added. After adding catalytic amount of DMF (0.1 mL) the solution was stirred at room temperature for 2 h. A solution of *o*-phenylenediamine (0.8 g, 7.5 mmol) and diisopropylethylamine (2 g, 0.015 mol, 2.69 mL) was added dropwise and the resulting solution was stirred at room temperature for 18 h. After removal of all volatiles the resulting residue was dissolved in EtOH (50 mL) and treated with hydrochloric acid (15 mL, 1 M). The resulting clear solution was stirred under reflux for 10 h, cooled to ambient temperature

and the deposited white solid was filtered and washed with cold ethanol (3 x 5 mL, -10 °C) and dried in vacuum to afford **23** as white solid. Yield: 1.9 g (65%). MS (HRESI): *m/z* = 1254.5433 $[M+H]^+$, 1276.5260 $[M+Na]^+$. ^{1}H NMR (400 MHz, DMSO-d_6) δ 11.64 (s, 1H), 10.47 (s, 1H), 7.84 – 7.78 (m, 4H), 7.35 – 7.29 (m, 4H), 7.01 (dd, J = 9.0, 4.6 Hz, 2H). ^{13}C NMR (101 MHz, DMSO-d_6) δ 165.34, 156.64, 154.78, 154.31, 131.34, 126.34, 125.76, 121.27 (d, J = 23.5 Hz), 119.16 (d, J = 7.6 Hz), 118.31, 115.63 (d, J = 24.5 Hz). ^{19}F NMR (376 MHz, DMSO-d_6) δ -124.29 (m).

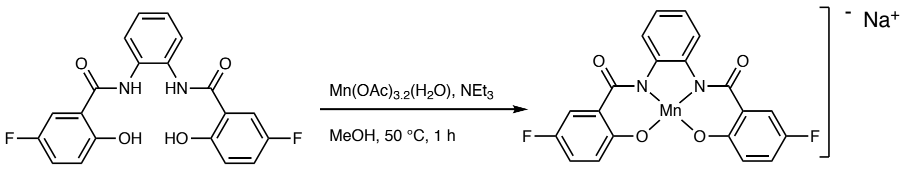

23 62% 24

MnL1F, **24**. Compound **23** (40 mg, 0.14 mmol) was dissolved in methanol (5 mL) and $Mn(OAc)_3.2H_2O$ (26 mg, 0.1 mmol) was added. The mixture was stirred at 60 °C for 5 min and the resulting clear brown solution was treated with NaOH (19 mg, 0.5 mmol). The solution was stirred at 60 °C for additional 30 min and the solvent was removed in the vacuum. The resulting solid was washed with DCM (3 x 5 mL) and cold water (1 mL, 5 °C) and dried in vacuum to afford **MnL1F** (**24**) as brown solid. Yield: 27 mg (62%).

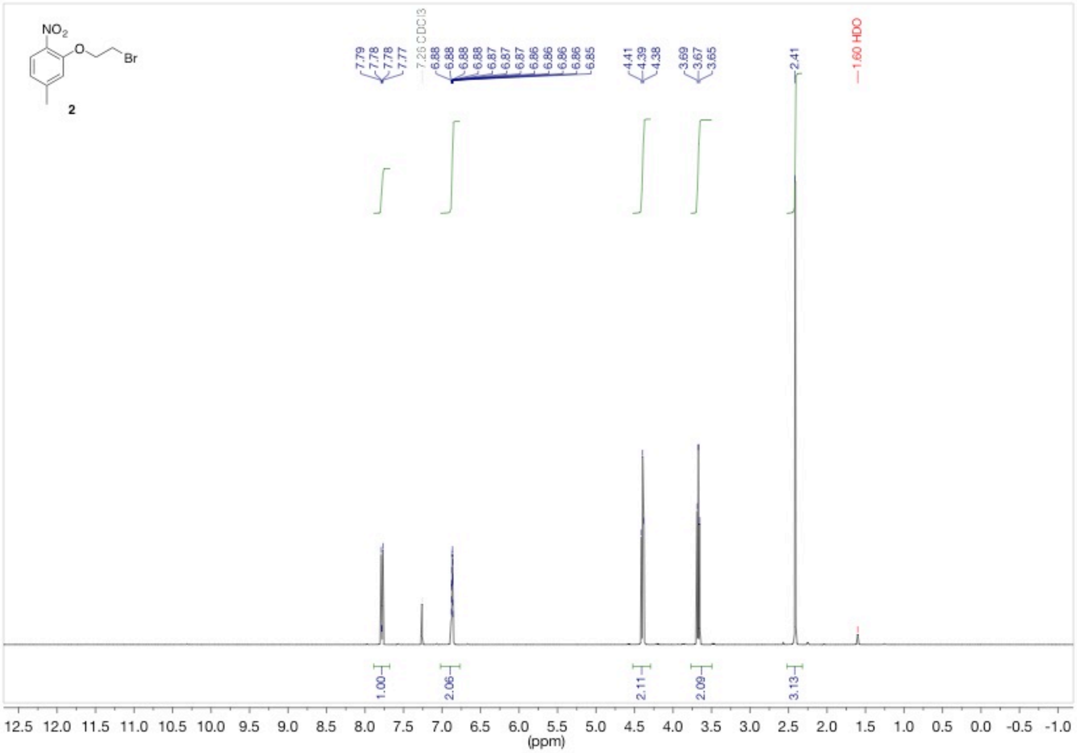

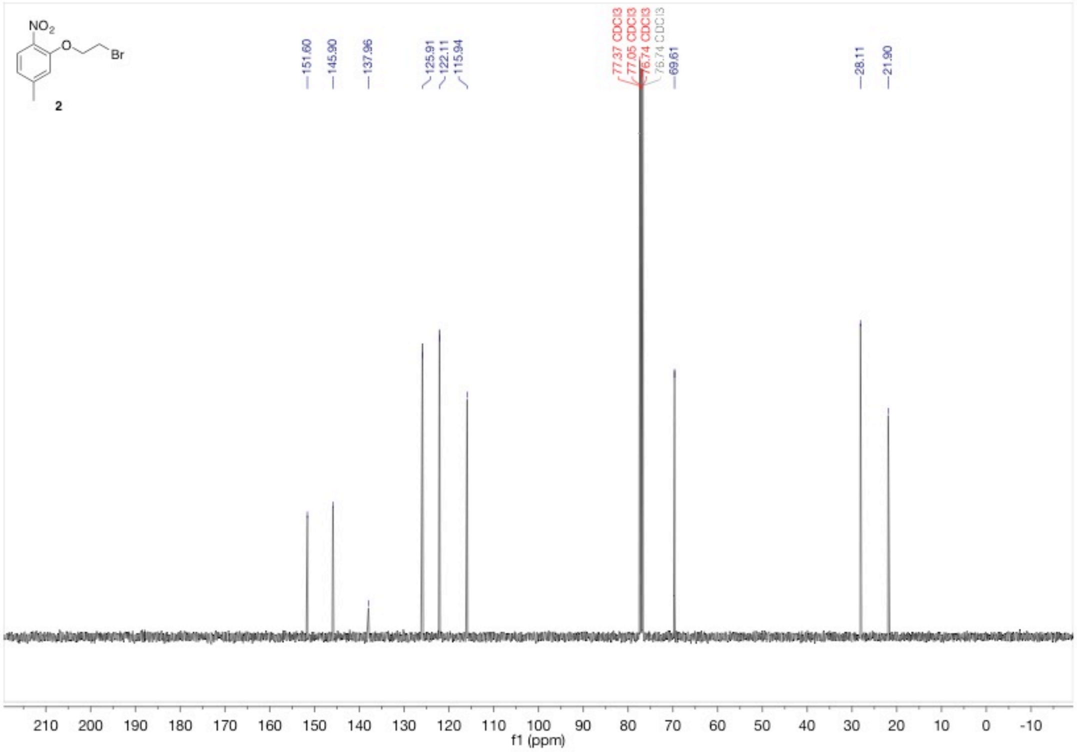

Supplementary Figure 8. ^{1}H (top) and ^{13}C (bottom) NMR spectra of 2.

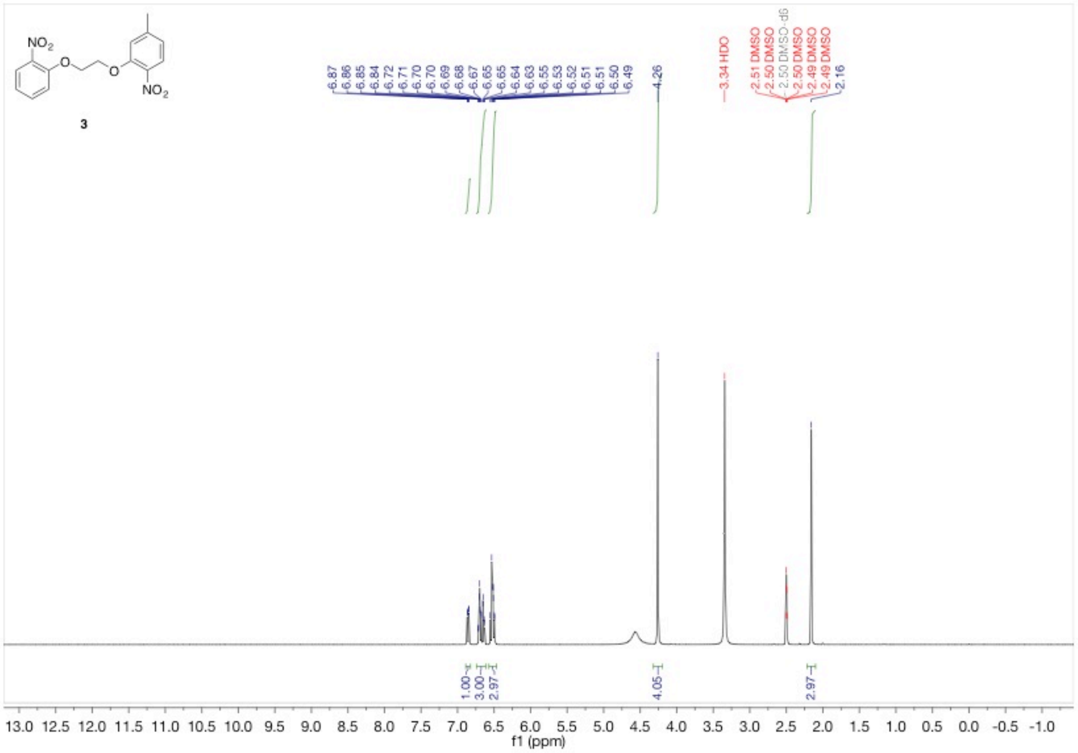

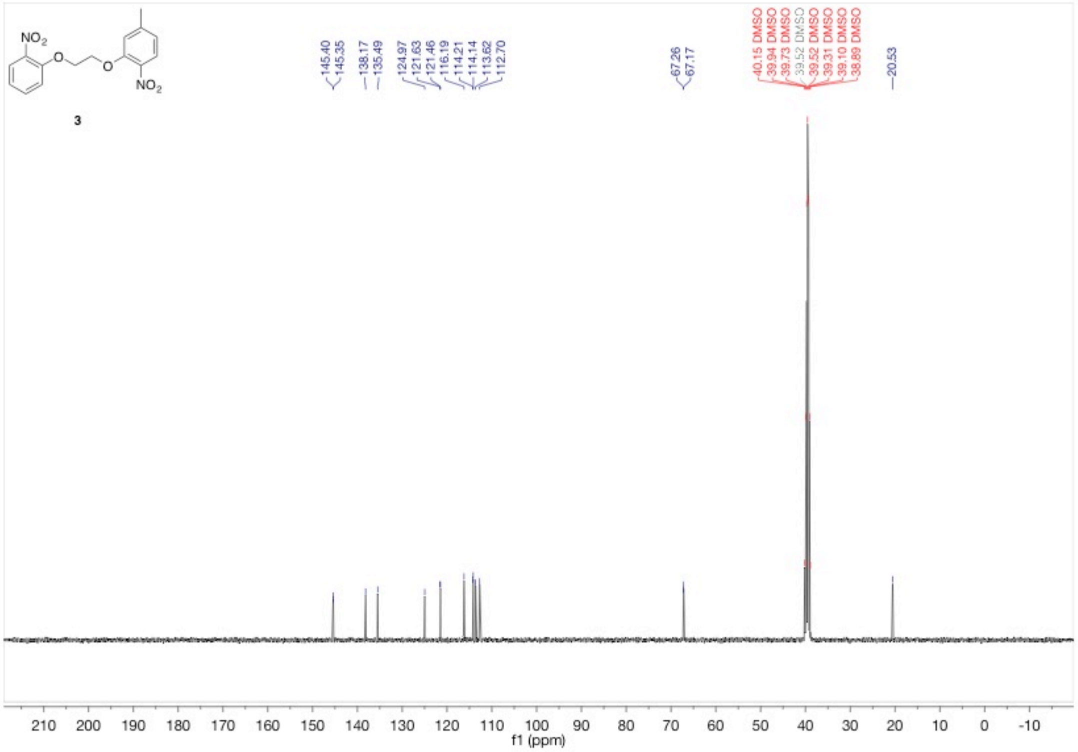

Supplementary Figure 9. ^{1}H (top) and ^{13}C (bottom) NMR spectra of 3.

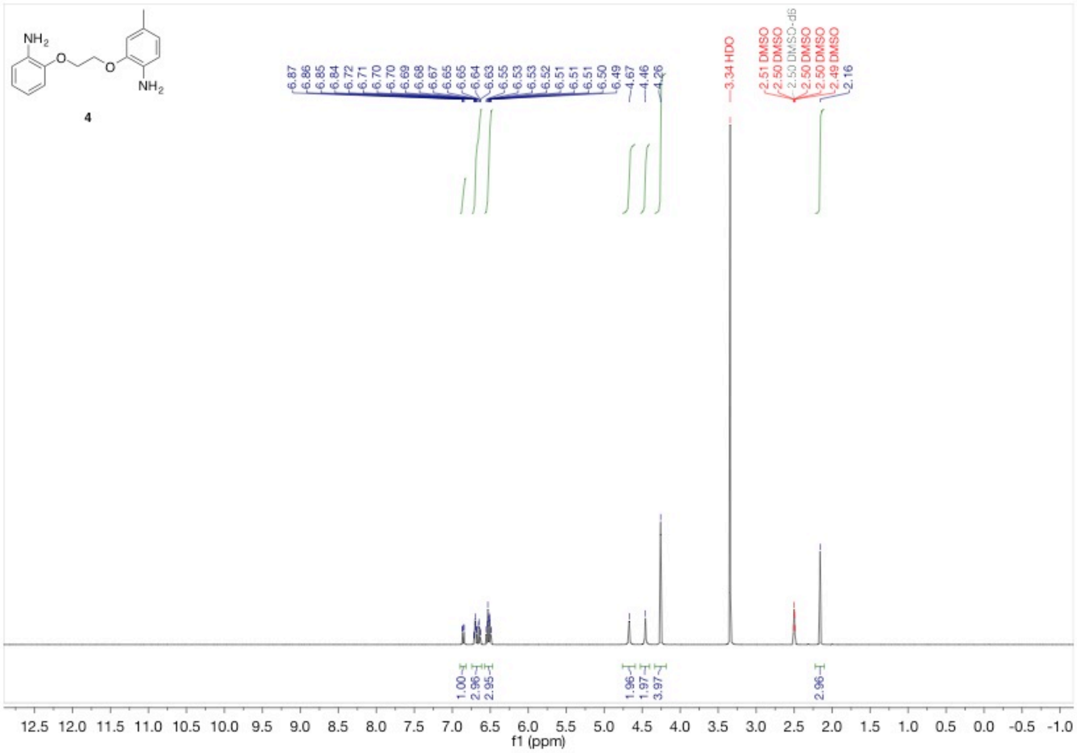

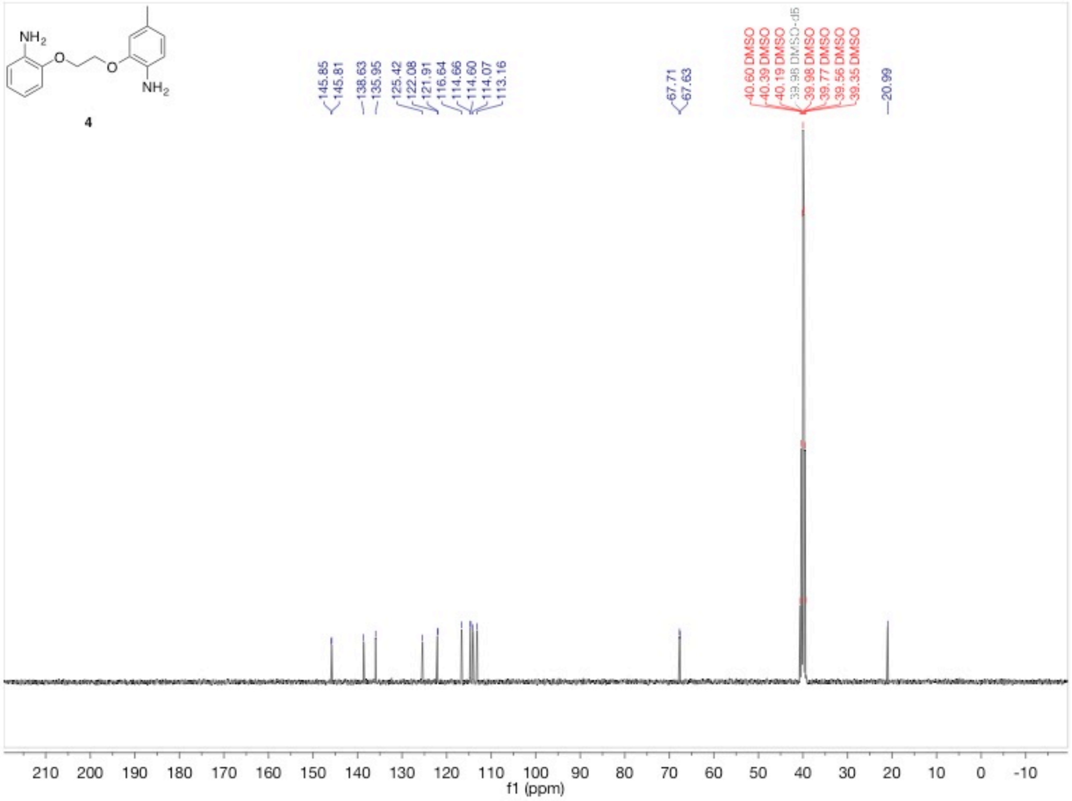

Supplementary Figure 10. ^{1}H (top) and ^{13}C (bottom) NMR spectra of 4.

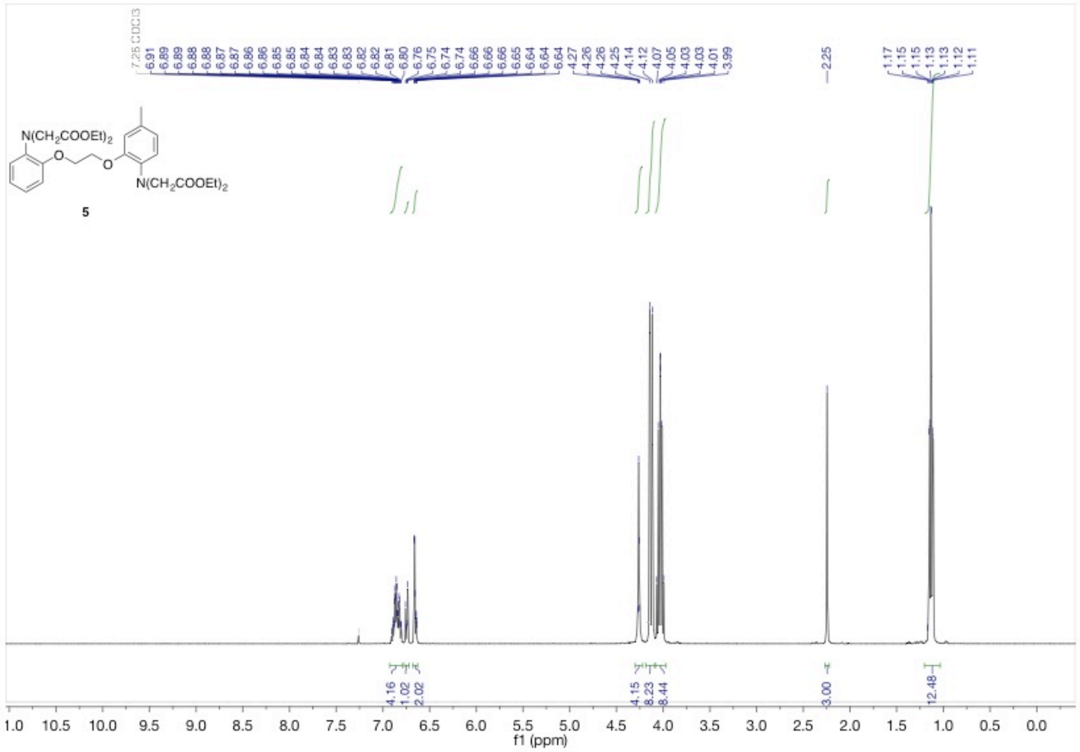

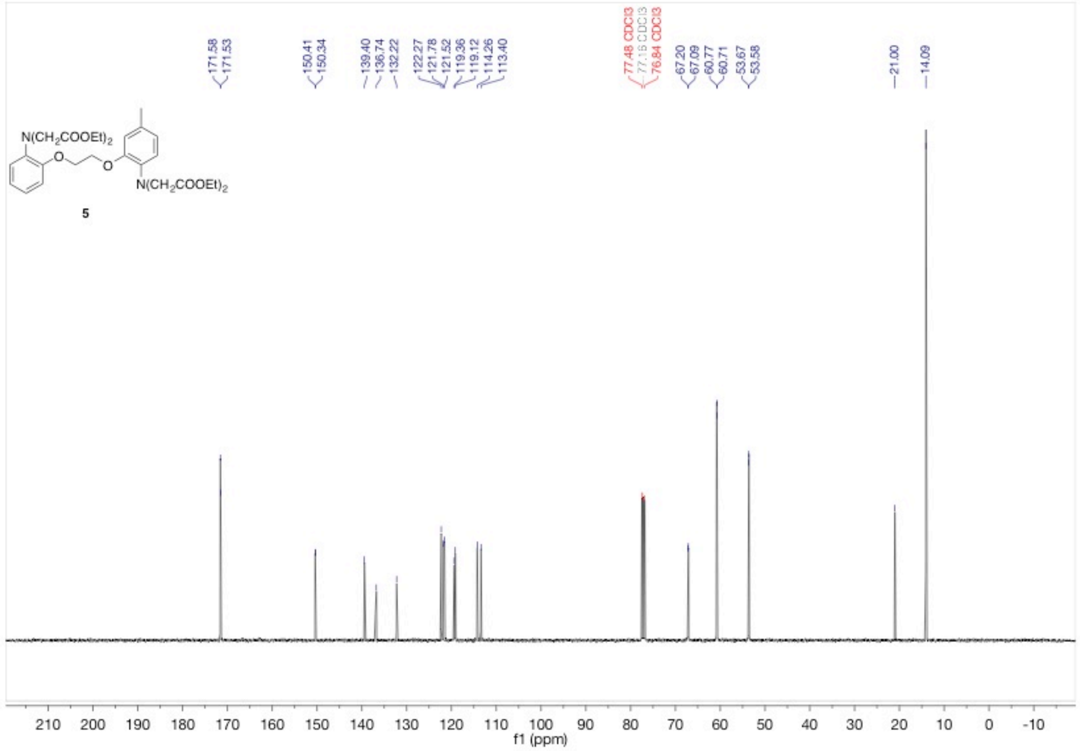

Supplementary Figure 11. ^{1}H (top) and ^{13}C (bottom) NMR spectra of 5.

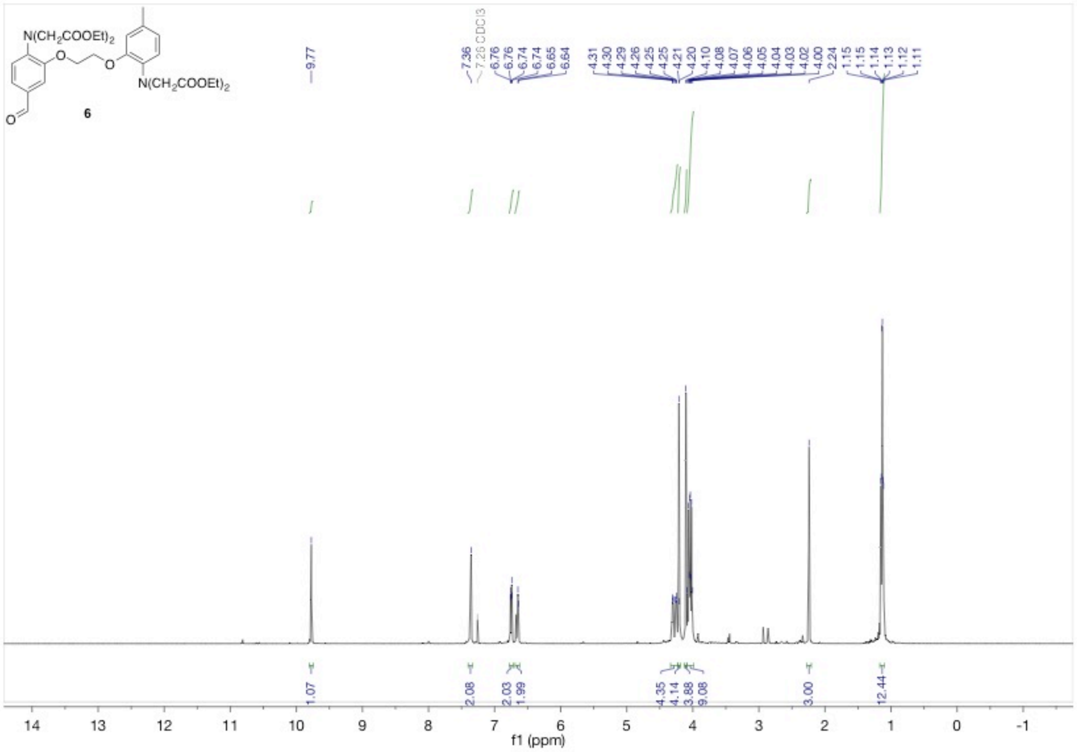

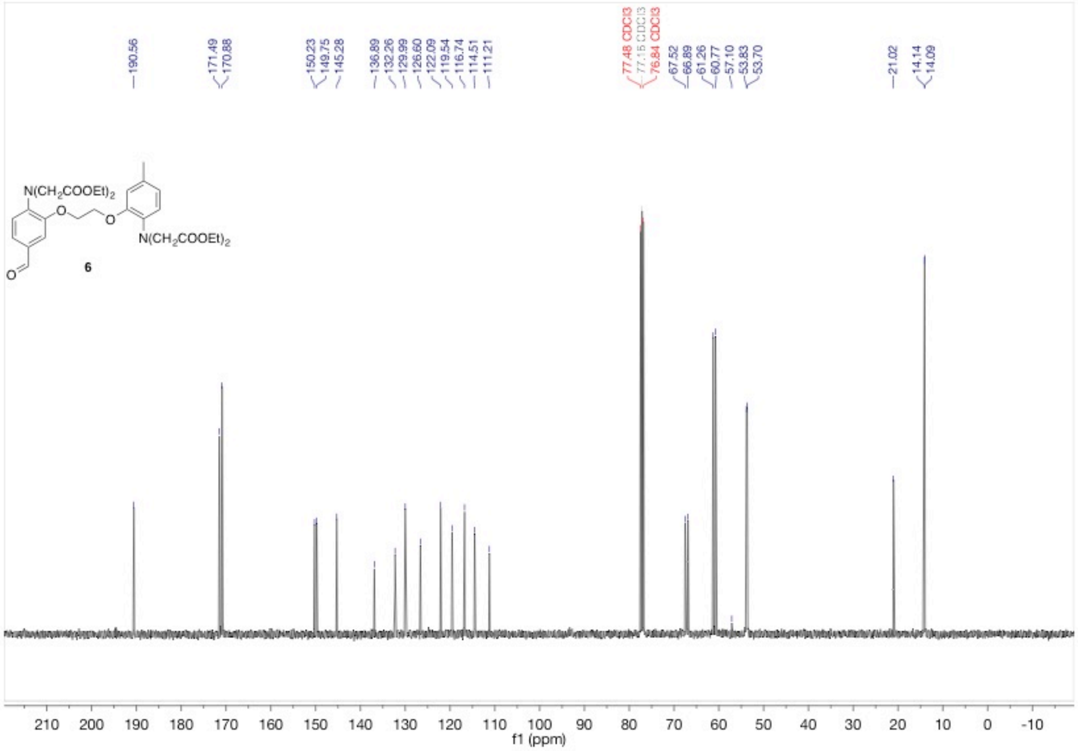

Supplementary Figure 12. ^{1}**H (top) and** ^{13}**C (bottom) NMR spectra of 6.**

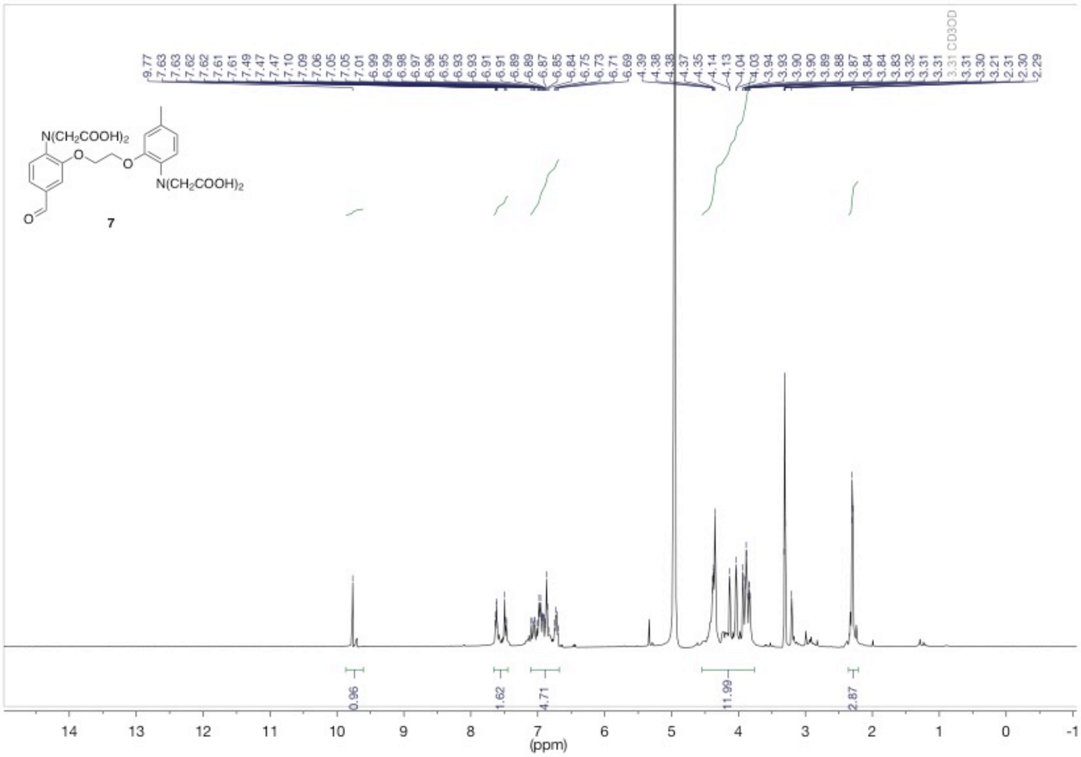

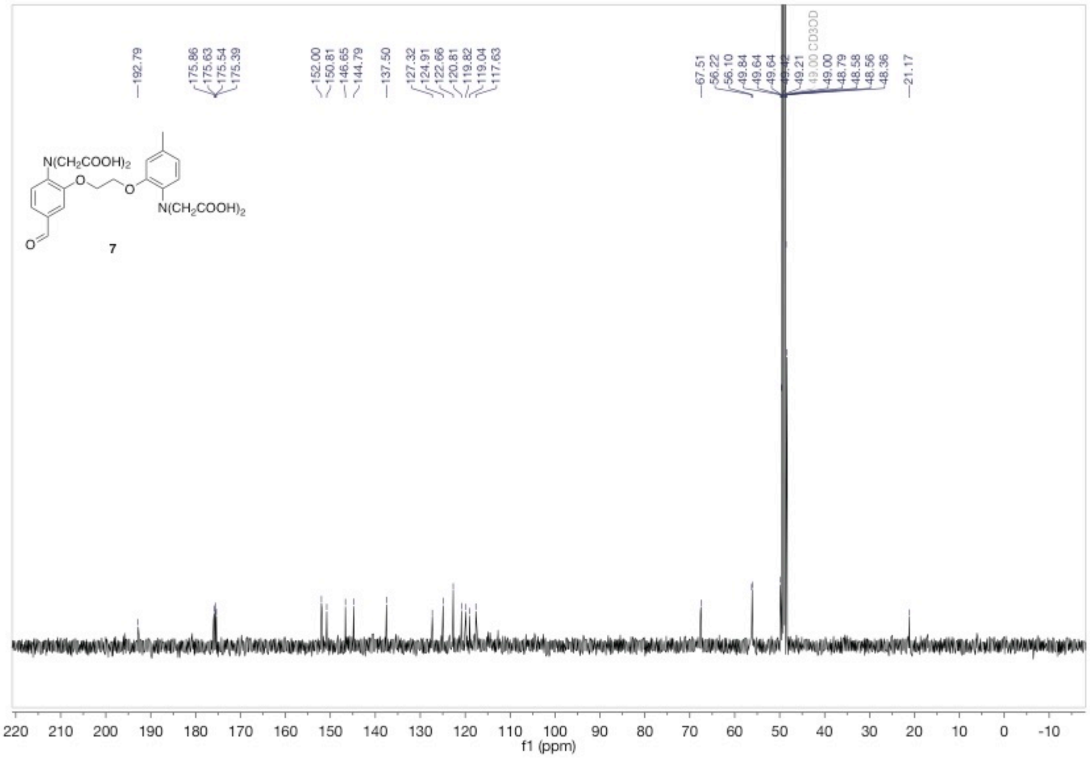

Supplementary Figure 13. ^{1}H (top) and ^{13}C (bottom) NMR spectra of 7.

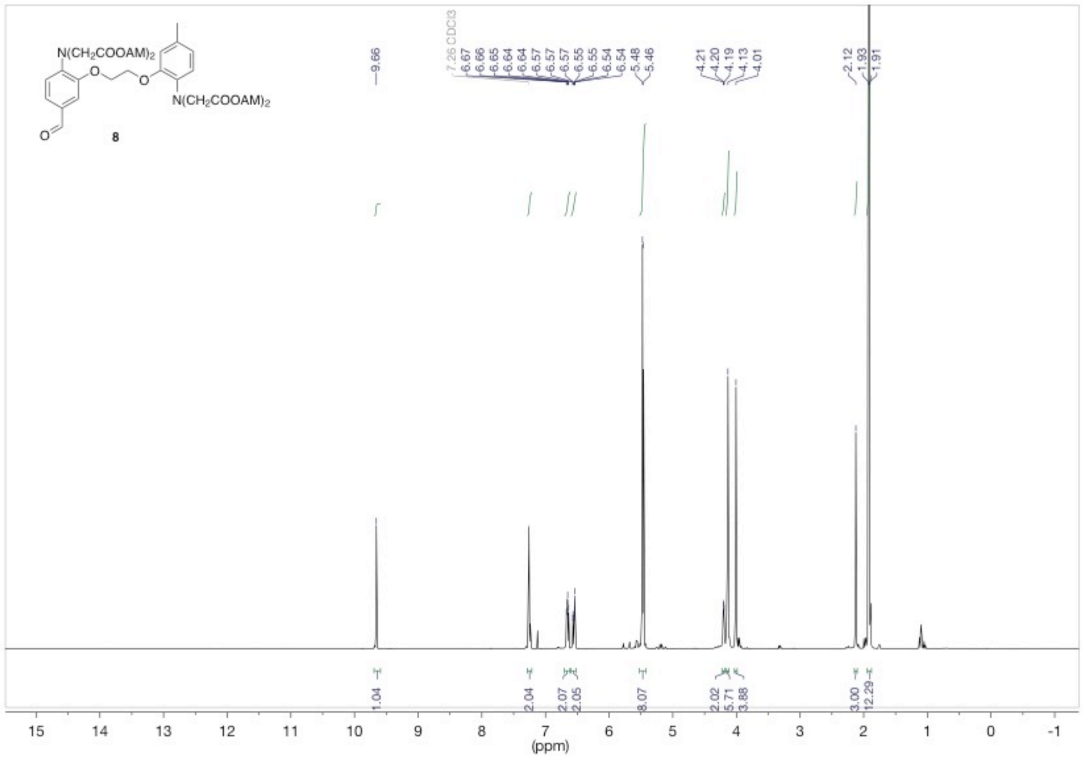

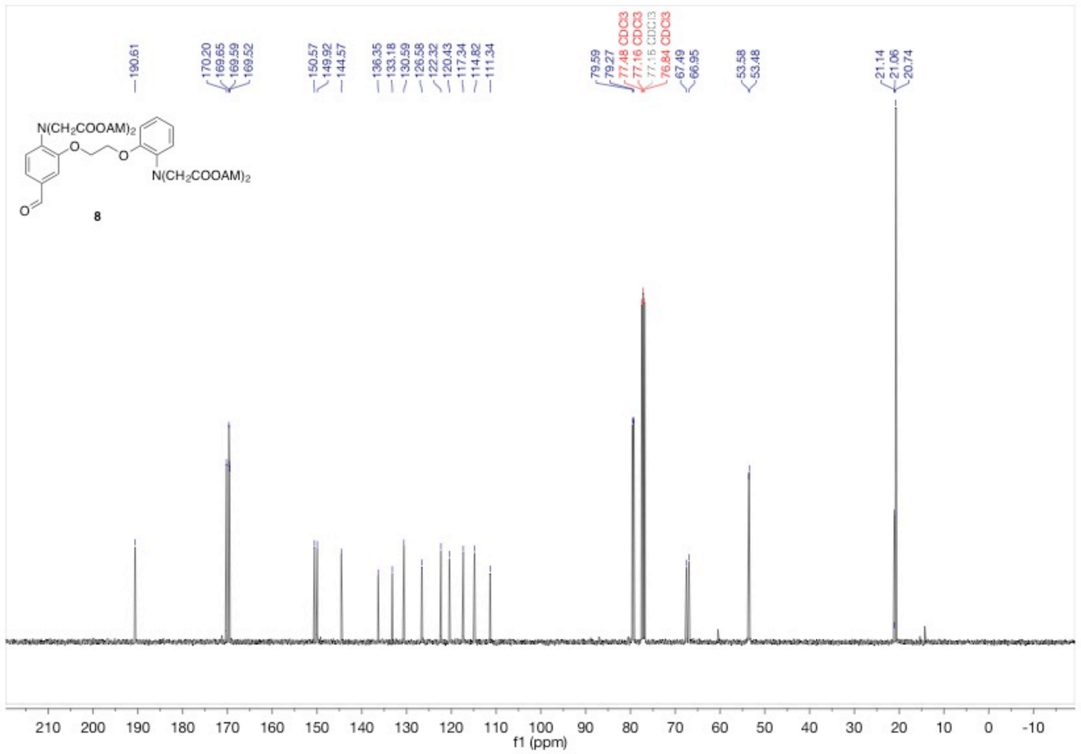

Supplementary Figure 14. ^{1}H (top) and ^{13}C (bottom) NMR spectra of 8.

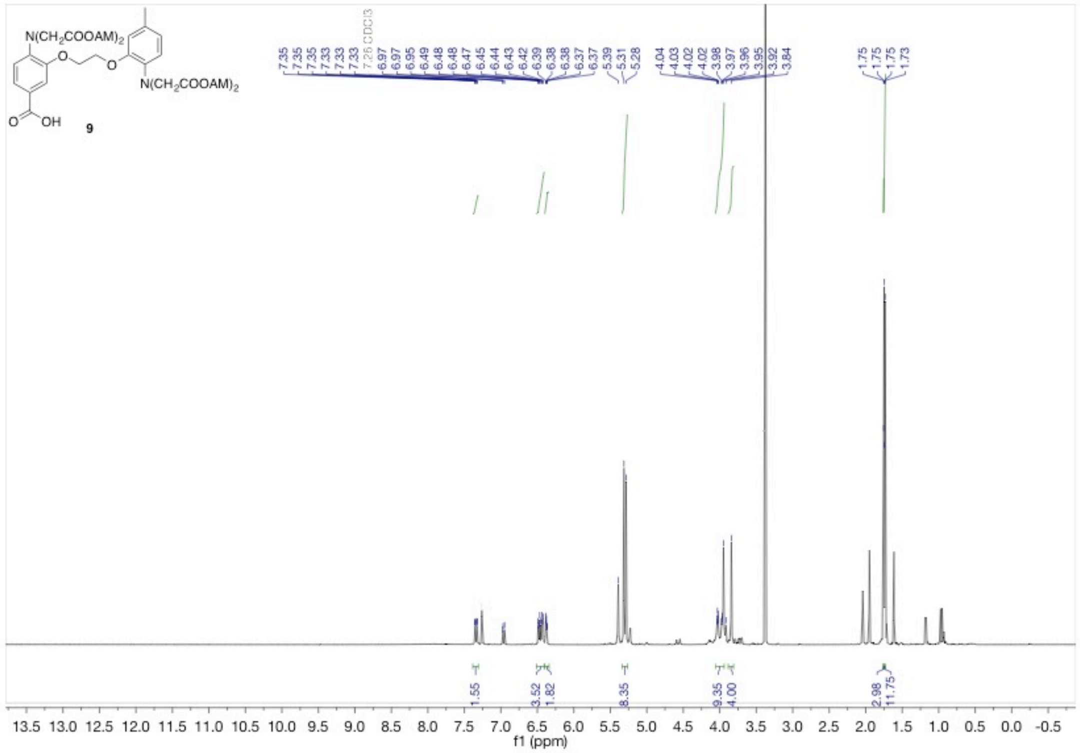

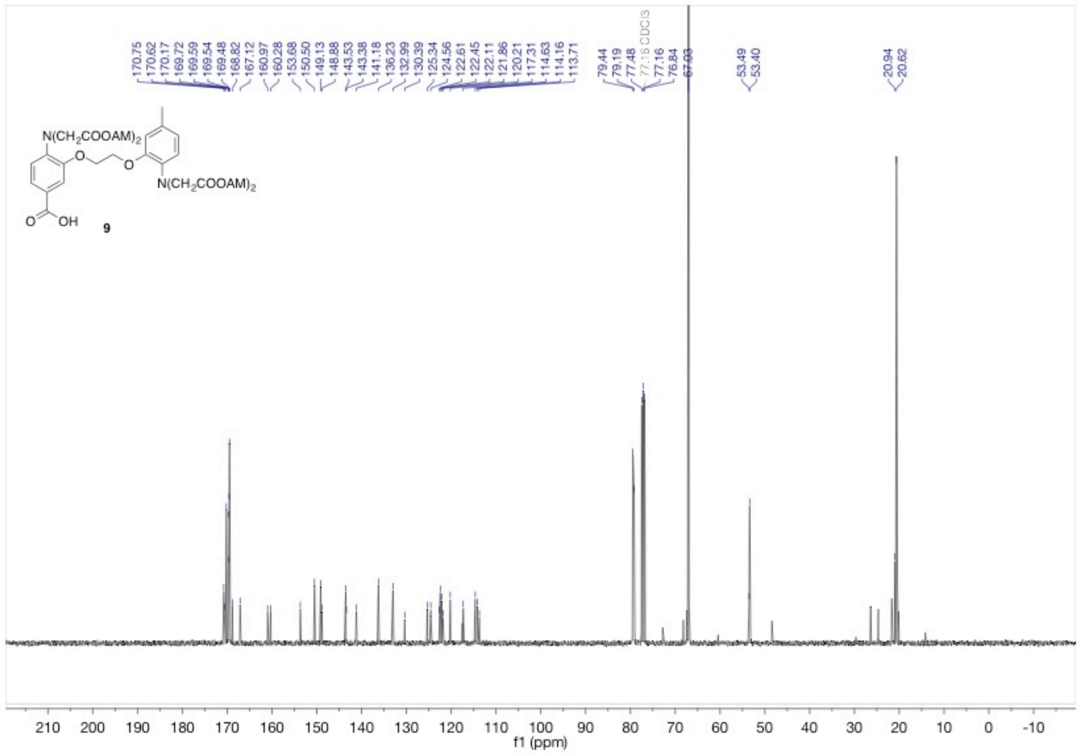

Supplementary Figure 15. ^{1}H (top) and ^{13}C (bottom) NMR spectra of 9.

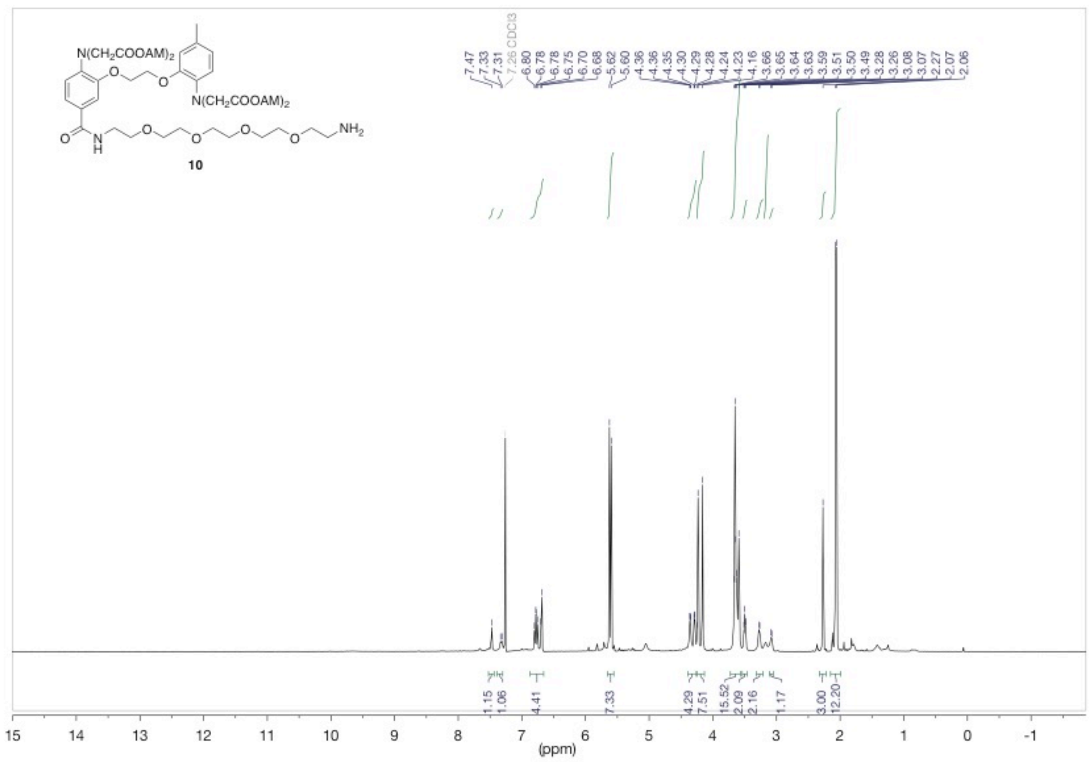

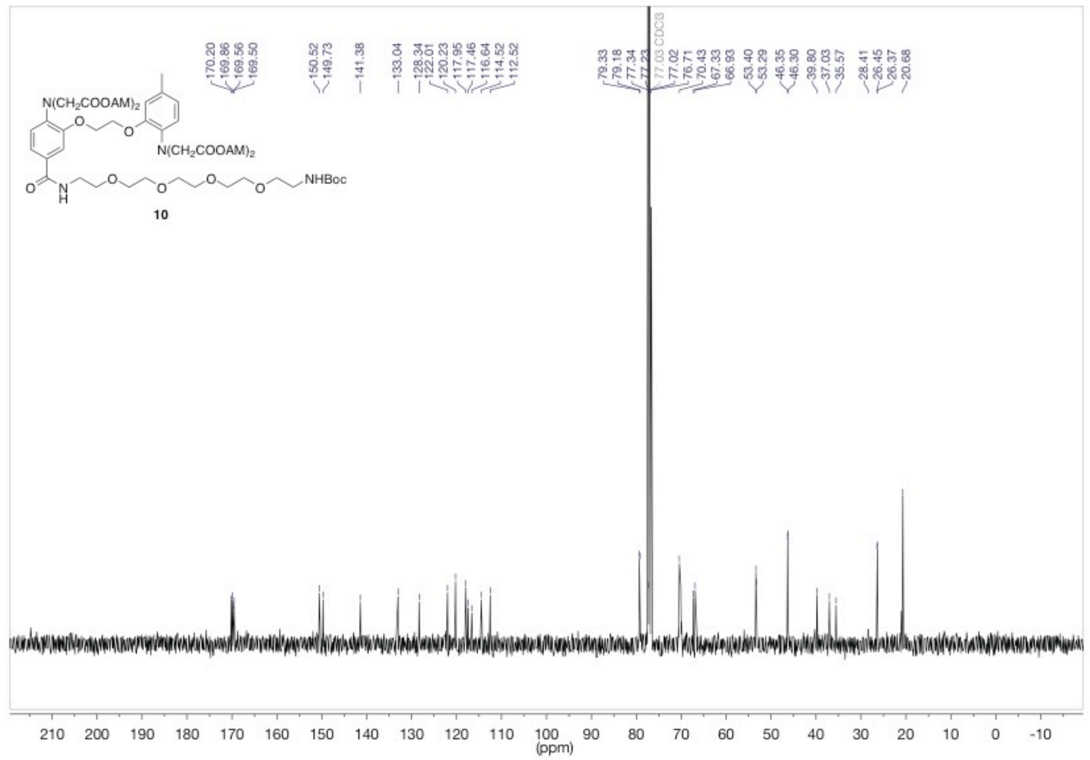

Supplementary Figure 16. ^{1}**H (top) and** ^{13}**C (bottom) NMR spectra of 10.**

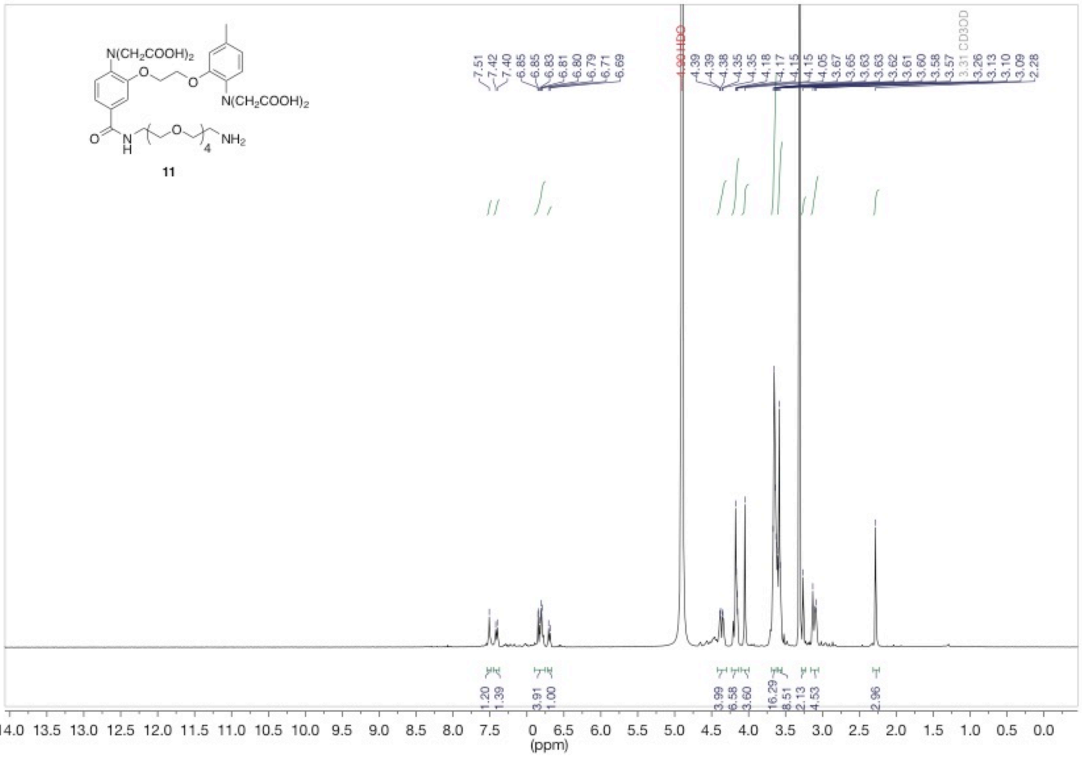

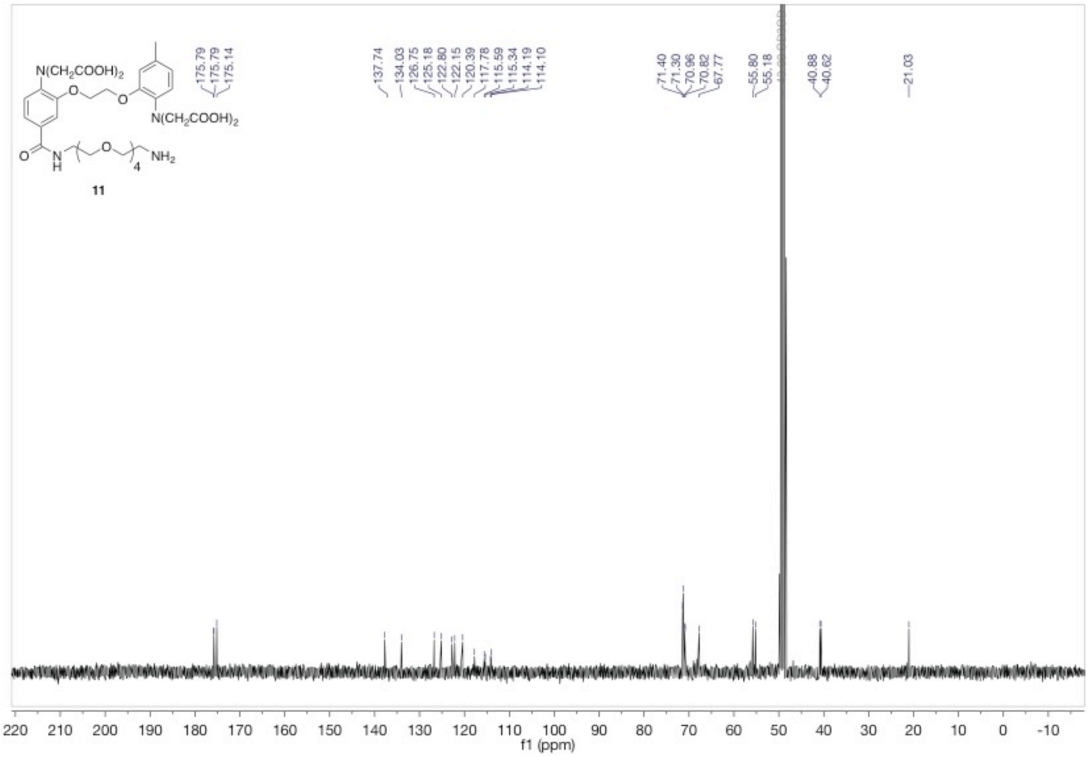

Supplementary Figure 17. ^{1}H (top) and ^{13}C (bottom) NMR spectra of 11.

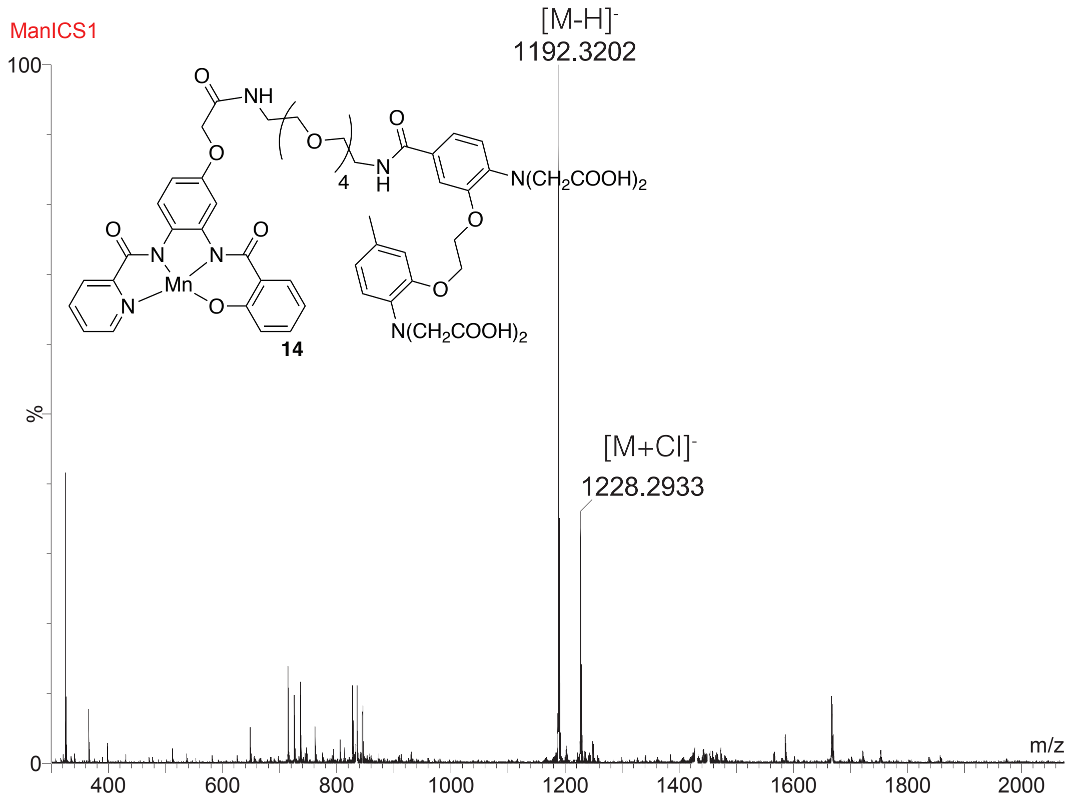

Supplementary Figure 18. Mass spectrum of ManICS1 (14).

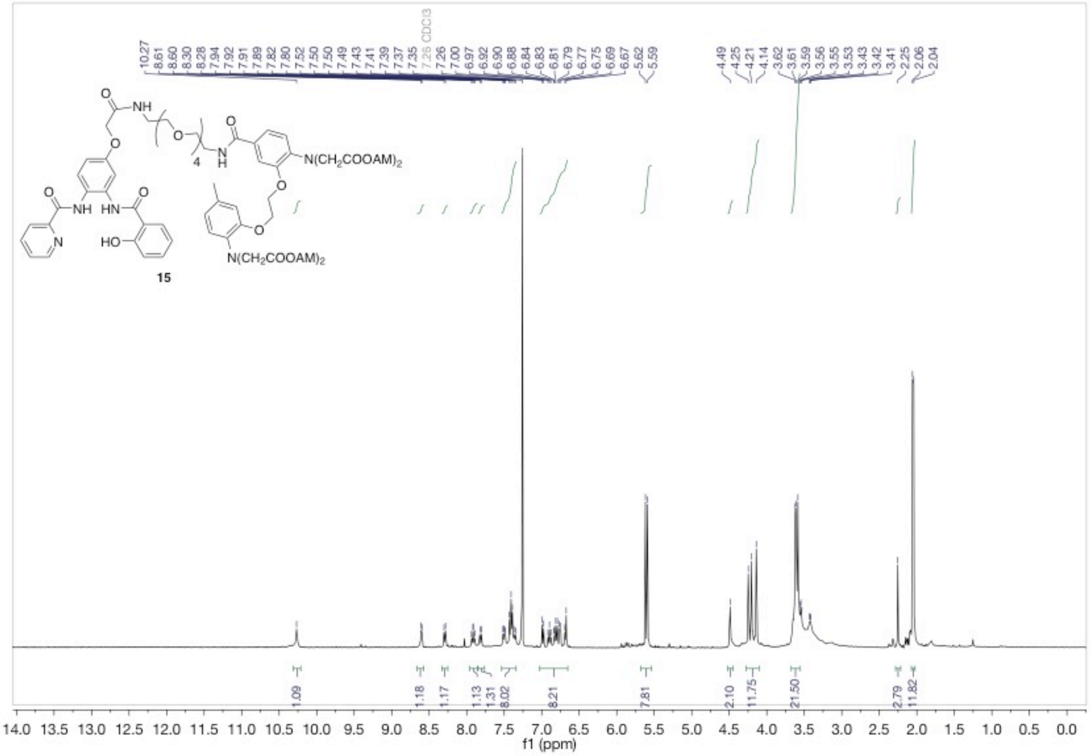

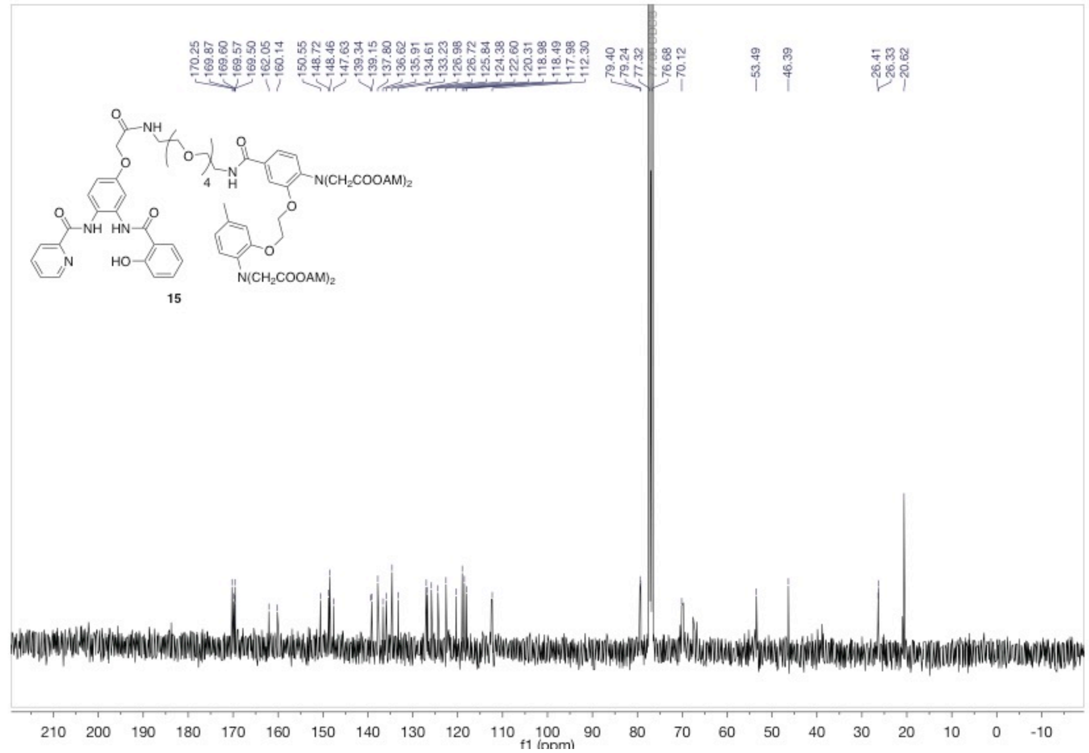

Supplementary Figure 19. ^{1}H (top) and ^{13}C (bottom) NMR spectra of 15.

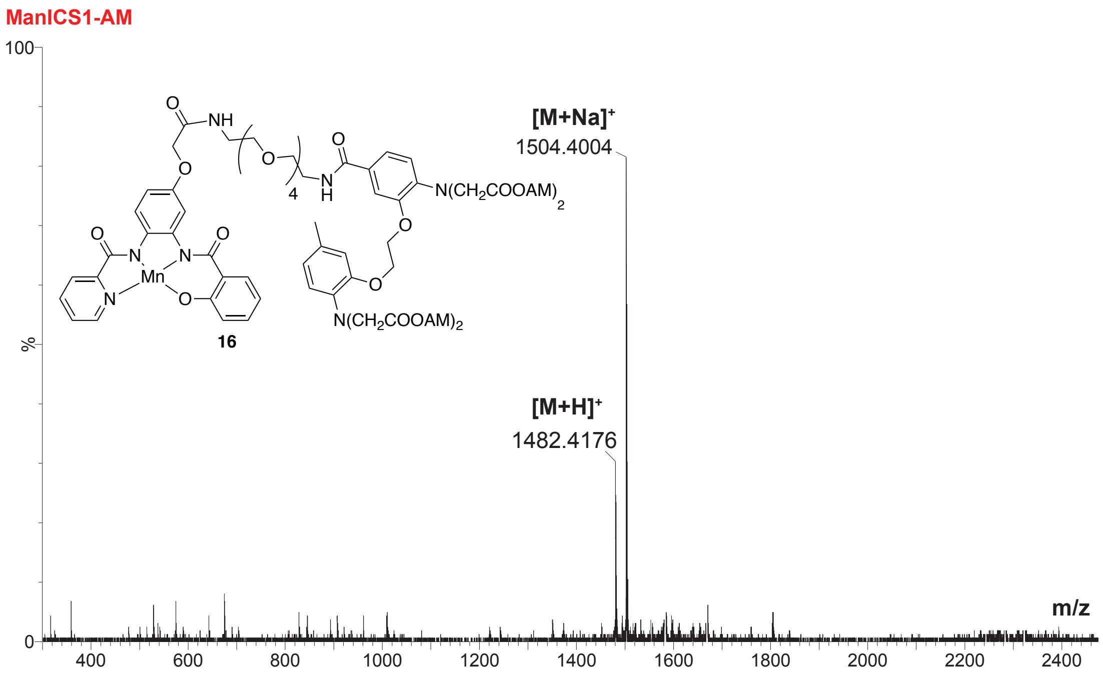

Supplementary Figure 20. Mass spectrum of ManICS1-AM (16).

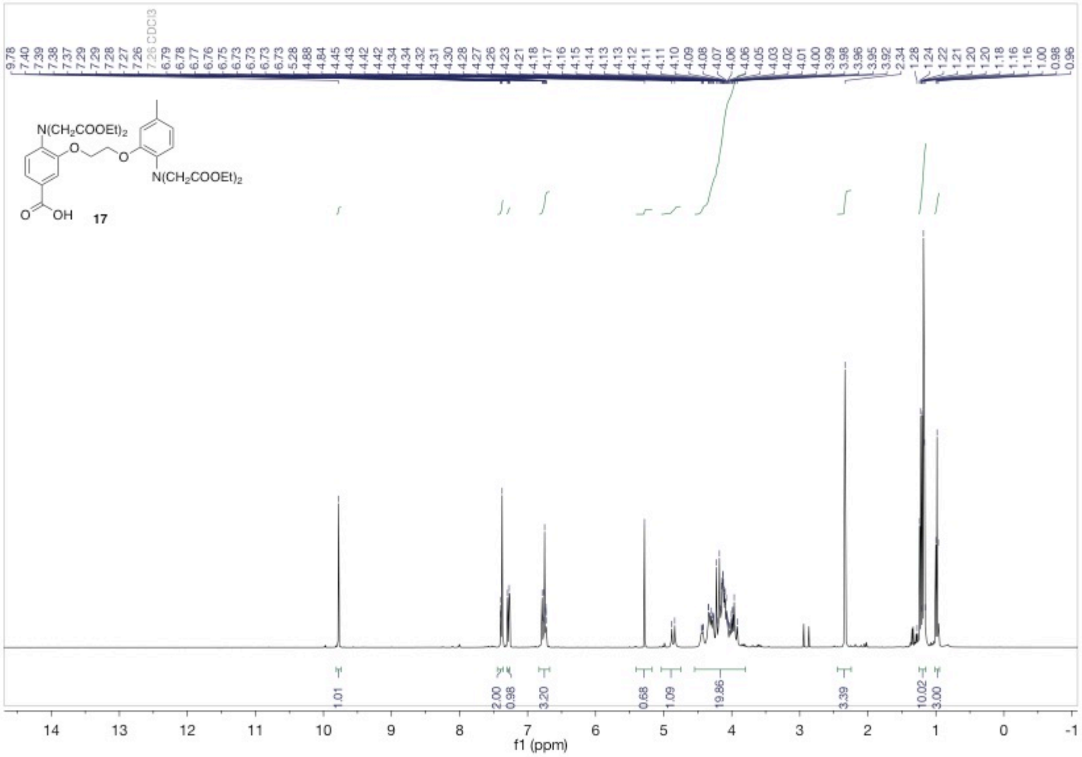

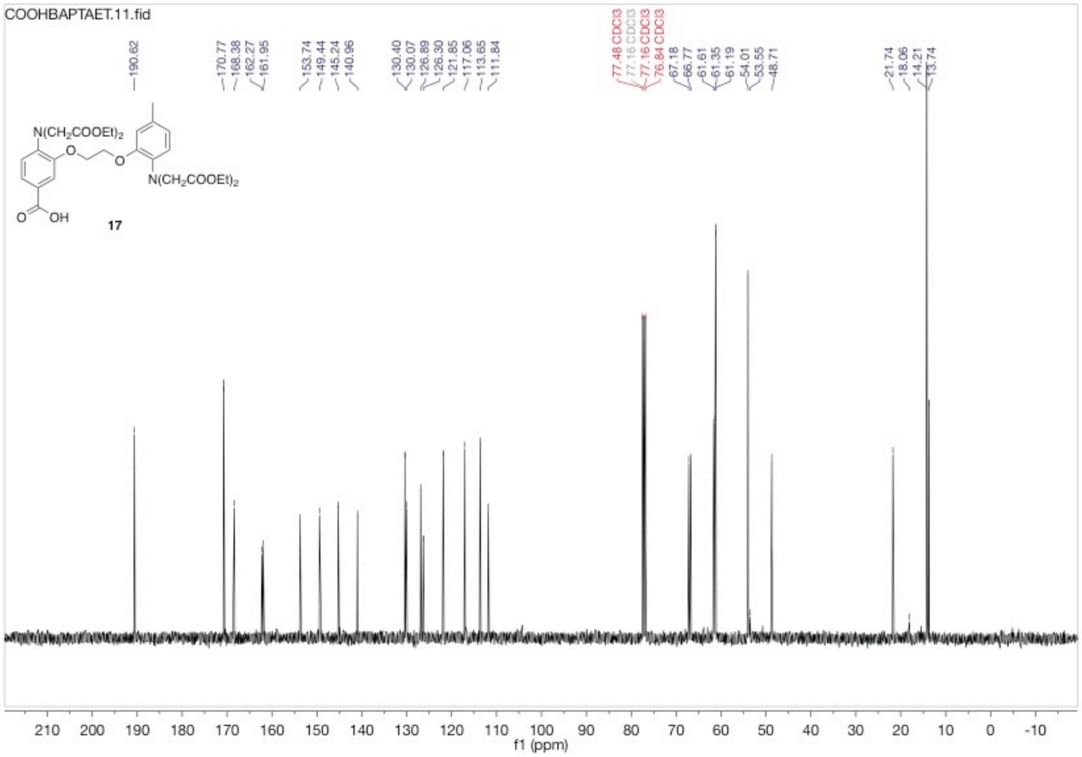

Supplementary Figure 21. ^{1}H (top) and ^{13}C (bottom) NMR spectra of 17.

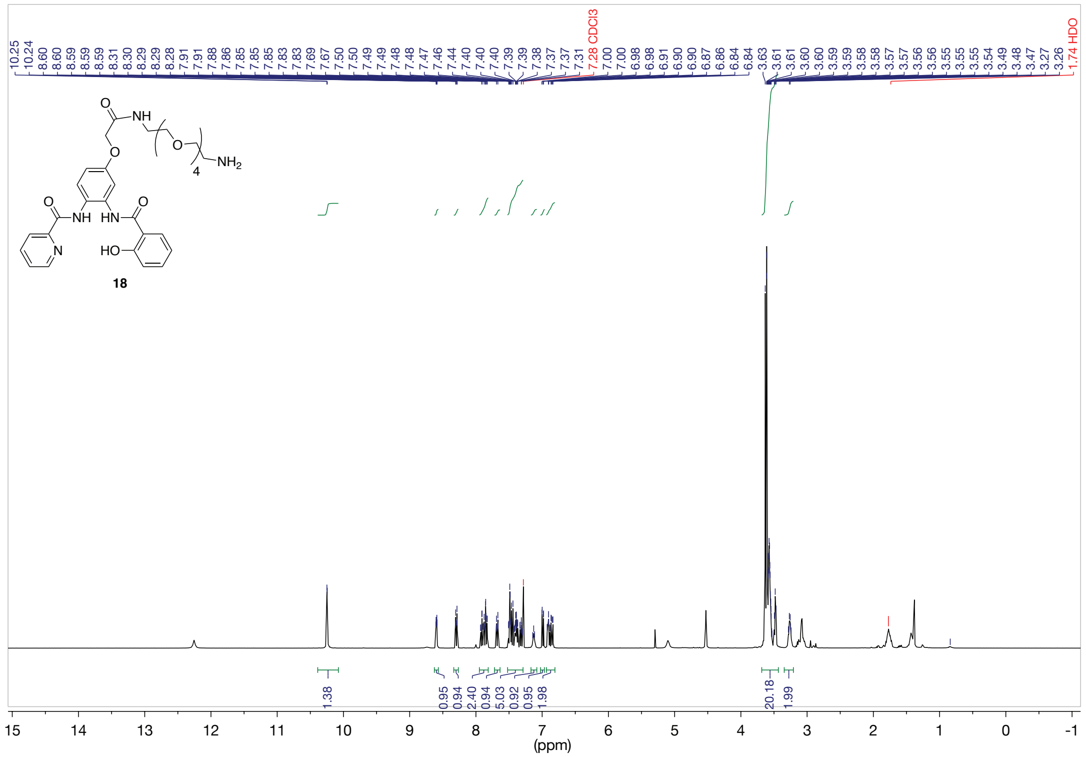

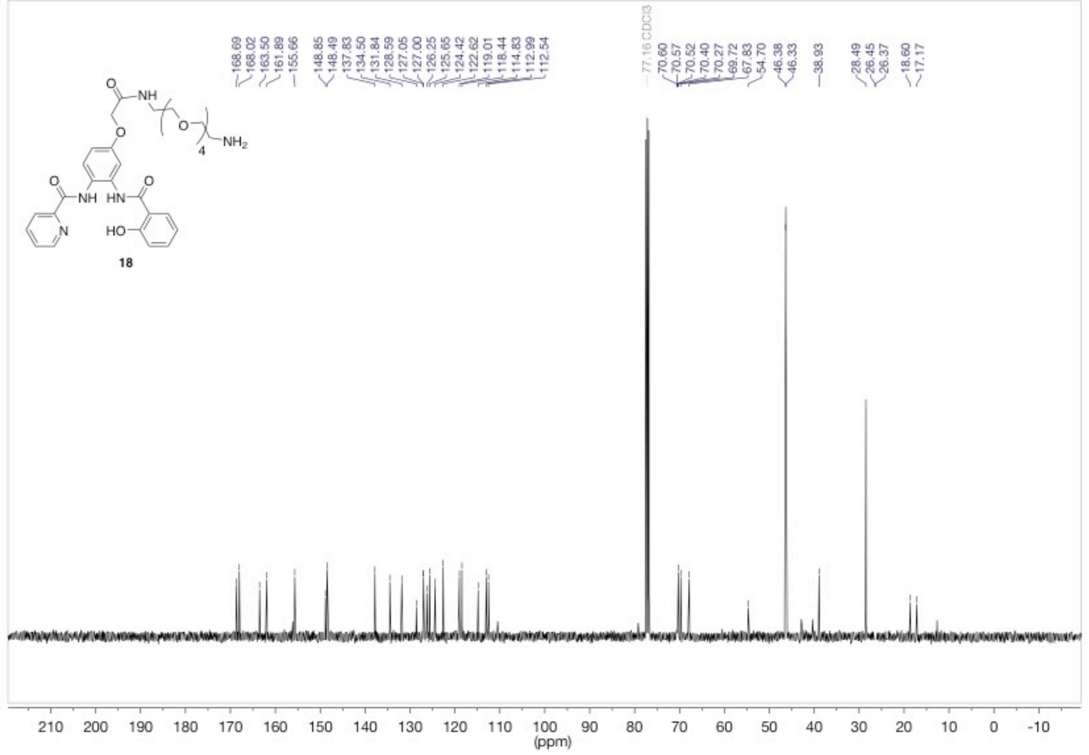

Supplementary Figure 22. ^{1}H (top) and ^{13}C (bottom) NMR spectra of 18.

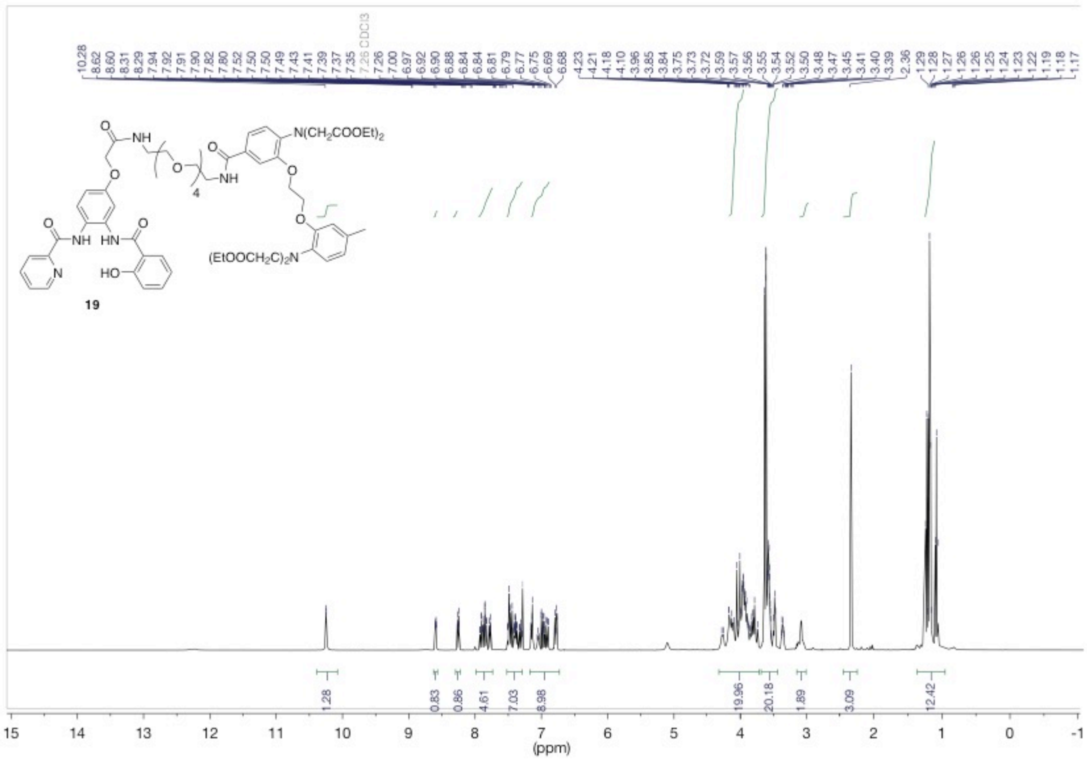

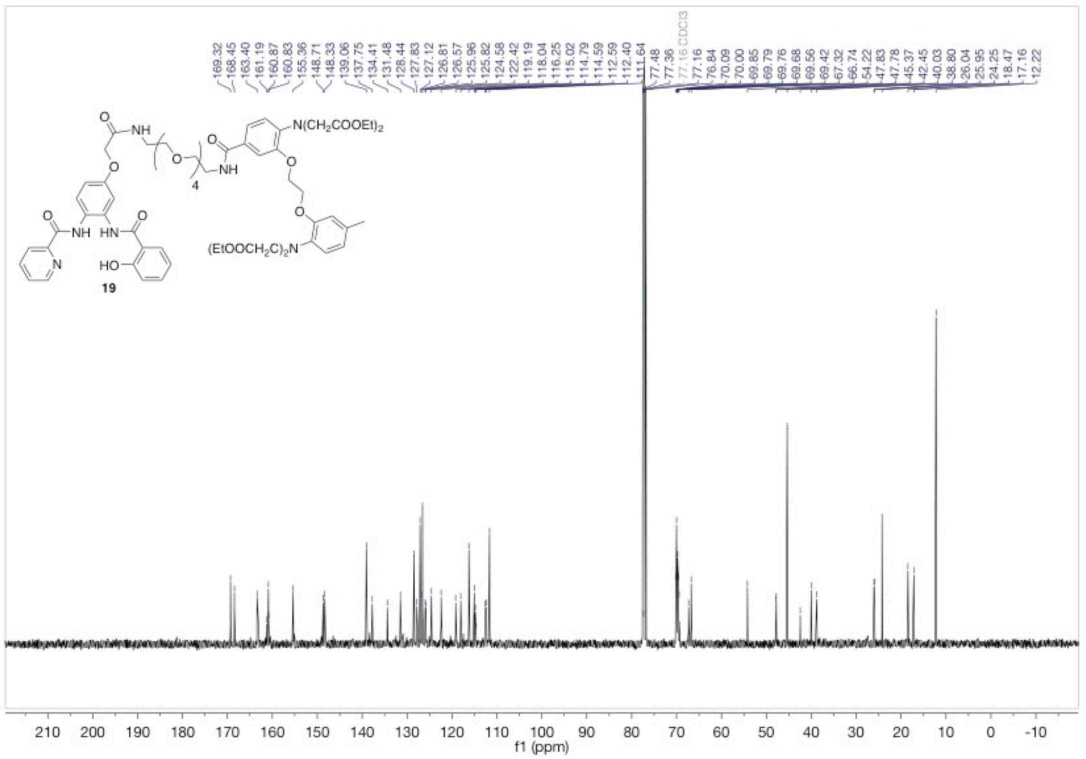

Supplementary Figure 23. ^{1}H (top) and ^{13}C (bottom) NMR spectra of 19.

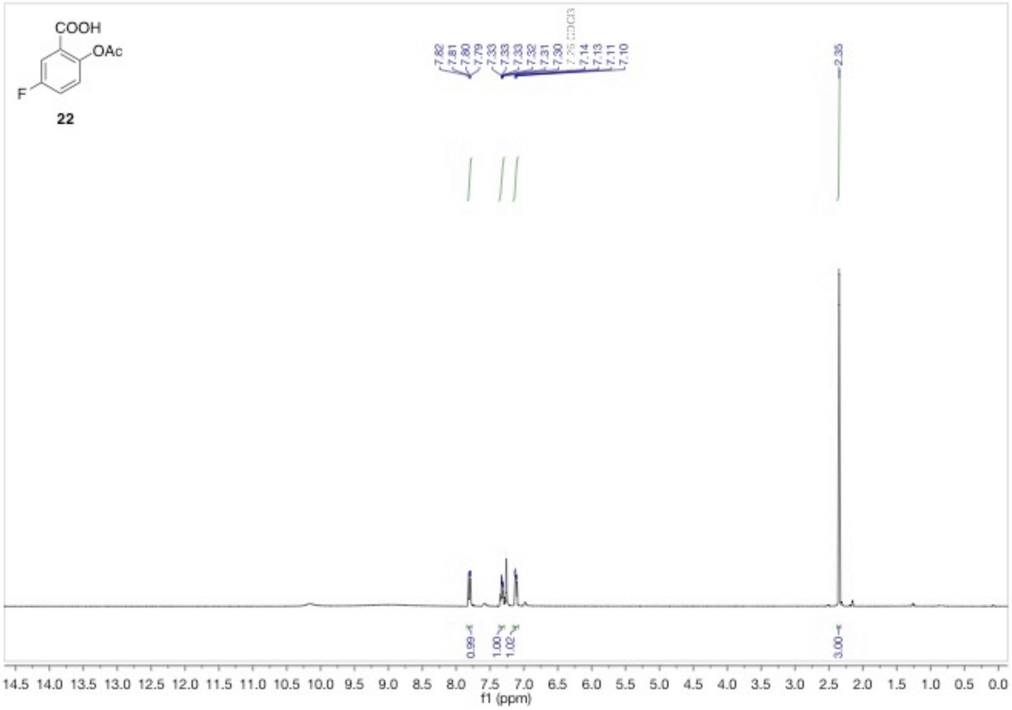

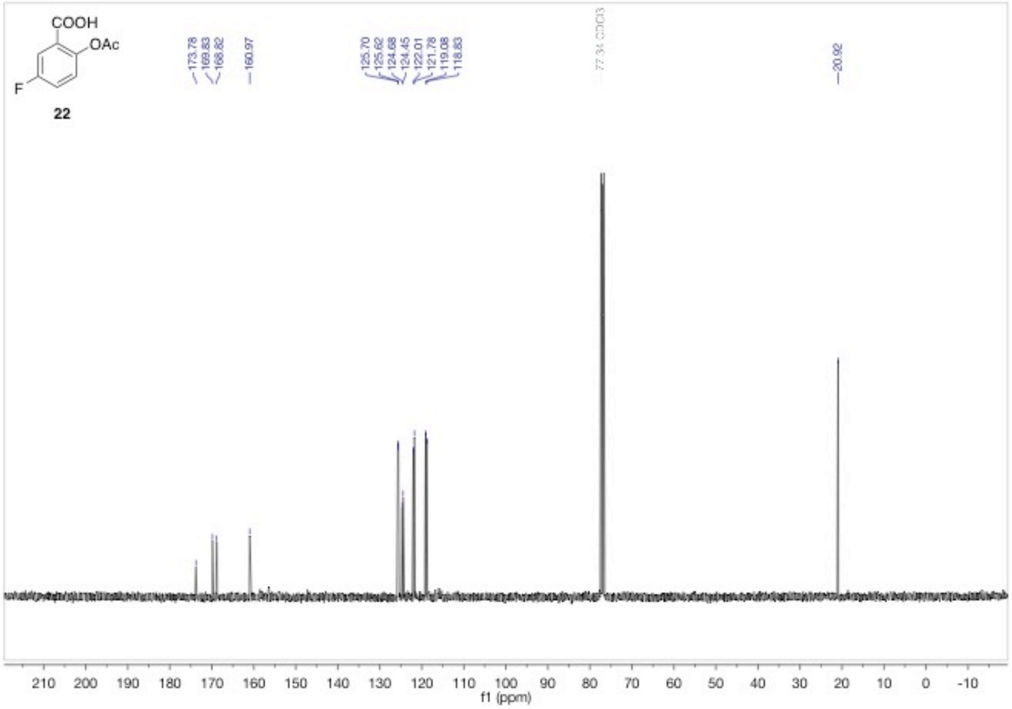

Supplementary Figure 24. ^{1}H (top) and ^{13}C (bottom) NMR spectra of 22.

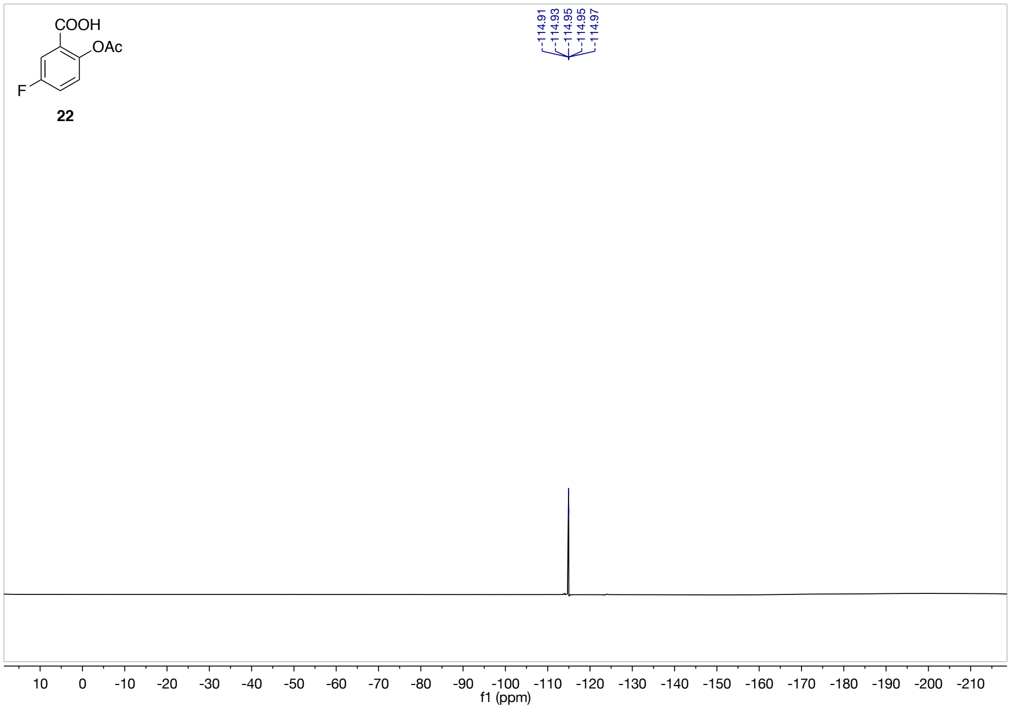

Supplementary Figure 25. ^{19}F NMR spectrum of 22.

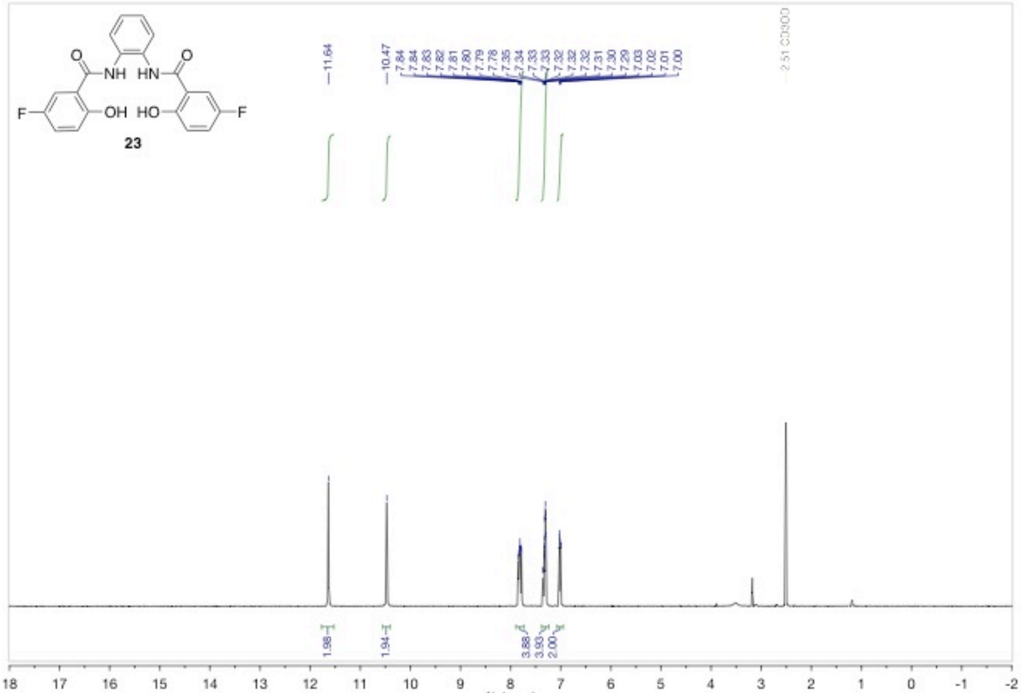

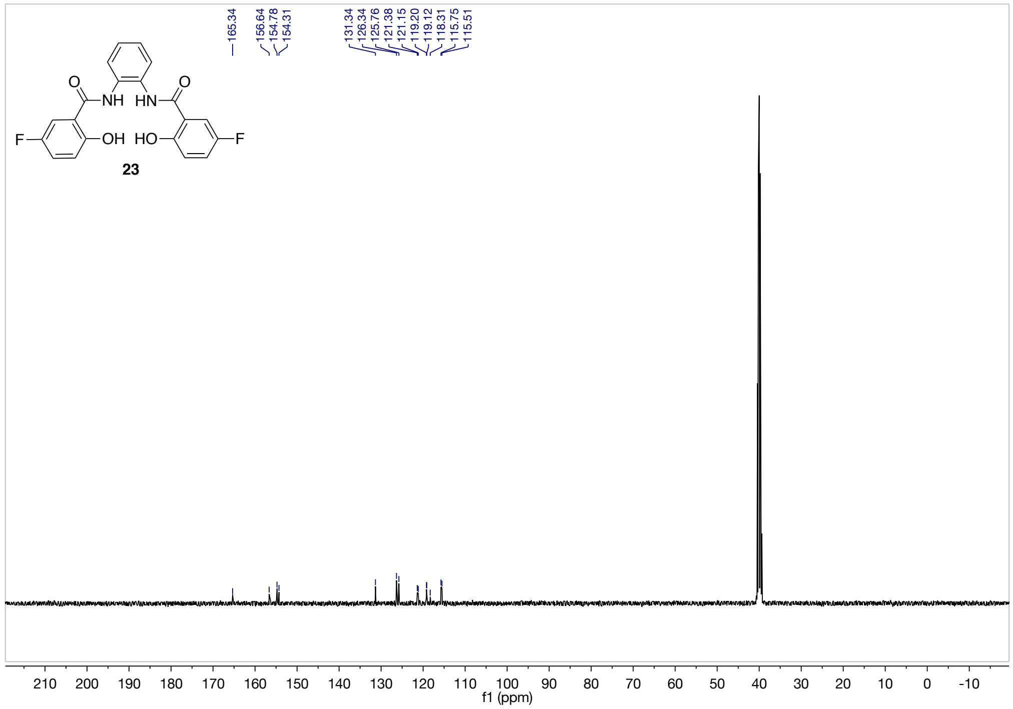

Supplementary Figure 26. ^{1}H (top) and ^{13}C (bottom) NMR spectra of 23.

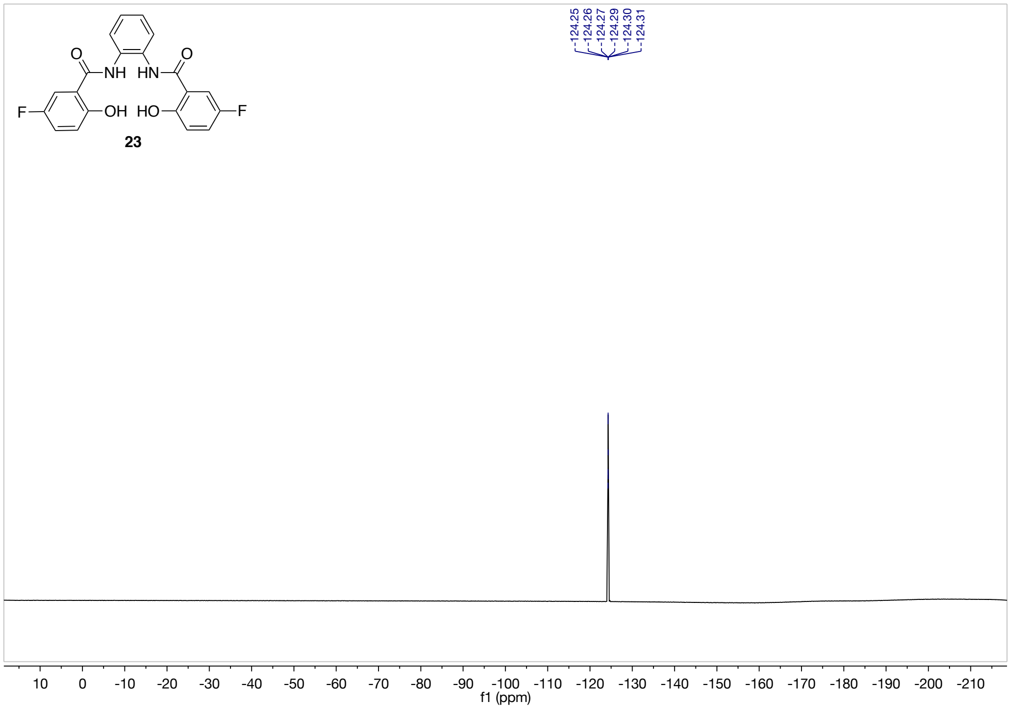

Supplementary Figure 27. ^{19}F NMR spectrum of 23.
