# Supplementary material for: Sensing intracellular calcium ions using a manganese-based MRI contrast agent
Source: Nat Commun. 2019 Feb 22;10:897. doi: 10.1038/s41467-019-08558-7 (PMC6385297; doi:10.1038/s41467-019-08558-7)
Supplement: Supplementary file 3 — Reporting Summary [file 41467_2019_8558_MOESM3_ESM.pdf]

nature research

Corresponding author(s): Alan Jasanoff

Last updated by author(s): Jan 4, 2019

Reporting Summary

Nature Research wishes to improve the reproducibility of the work that we publish. This form provides structure for consistency and transparency in reporting. For further information on Nature Research policies, see Authors & Referees and the Editorial Policy Checklist.

Statistics

For all statistical analyses, confirm that the following items are present in the figure legend, table legend, main text, or Methods section.

n/a	Confirmed	
☐	☒	The exact sample size (*n*) for each experimental group/condition, given as a discrete number and unit of measurement
☐	☒	A statement on whether measurements were taken from distinct samples or whether the same sample was measured repeatedly
☐	☒	The statistical test(s) used AND whether they are one- or two-sided<br>*Only common tests should be described solely by name; describe more complex techniques in the Methods section.*
☒	☐	A description of all covariates tested
☒	☐	A description of any assumptions or corrections, such as tests of normality and adjustment for multiple comparisons
☐	☒	A full description of the statistical parameters including central tendency (e.g. means) or other basic estimates (e.g. regression coefficient) AND variation (e.g. standard deviation) or associated estimates of uncertainty (e.g. confidence intervals)
☐	☒	For null hypothesis testing, the test statistic (e.g. *F*, *t*, *r*) with confidence intervals, effect sizes, degrees of freedom and *P* value noted<br>*Give P values as exact values whenever suitable.*
☒	☐	For Bayesian analysis, information on the choice of priors and Markov chain Monte Carlo settings
☒	☐	For hierarchical and complex designs, identification of the appropriate level for tests and full reporting of outcomes
☒	☐	Estimates of effect sizes (e.g. Cohen's *d*, Pearson's *r*), indicating how they were calculated

Our web collection on statistics for biologists contains articles on many of the points above.

Software and code

Policy information about availability of computer code

Data collection	Paravision (Bruker Instruments)
Data analysis	MATLAB (Mathworks), ImageJ (NIH); scripts used for data analysis are available upon reasonable request

For manuscripts utilizing custom algorithms or software that are central to the research but not yet described in published literature, software must be made available to editors/reviewers. We strongly encourage code deposition in a community repository (e.g. GitHub). See the Nature Research guidelines for submitting code & software for further information.

Data

Policy information about availability of data

All manuscripts must include a data availability statement. This statement should provide the following information, where applicable:

- Accession codes, unique identifiers, or web links for publicly available datasets
- A list of figures that have associated raw data
- A description of any restrictions on data availability

Raw MRI datasets generated during and/or analyzed during the current study are available from the corresponding author on reasonable request.

Field-specific reporting

Please select the one below that is the best fit for your research. If you are not sure, read the appropriate sections before making your selection.

☒ Life sciences ☐ Behavioural & social sciences ☐ Ecological, evolutionary & environmental sciences

For a reference copy of the document with all sections, see nature.com/documents/nr-reporting-summary-flat.pdf

Life sciences study design

All studies must disclose on these points even when the disclosure is negative.

Sample size	Sample sizes (n ≥ 5) used for each in vivo functional experiment were appropriate for providing a statistical power of 0.8 for detection of a ≥ 4% signal change over a standard deviation of 2%.
Data exclusions	Only experimental sessions that were prematurely terminated due to major technical failures were excluded from analysis.
Replication	All experiments were performed with multiple replicates, and standard statistical methods were used to accept or reject null hypotheses of no effect.
Randomization	n/a
Blinding	n/a

Reporting for specific materials, systems and methods

We require information from authors about some types of materials, experimental systems and methods used in many studies. Here, indicate whether each material, system or method listed is relevant to your study. If you are not sure if a list item applies to your research, read the appropriate section before selecting a response.

Materials & experimental systems

n/a	Involved in the study	
☒	☐	Antibodies
☐	☒	Eukaryotic cell lines
☒	☐	Palaeontology
☐	☒	Animals and other organisms
☒	☐	Human research participants
☒	☐	Clinical data

Methods

n/a	Involved in the study	
☒	☐	ChIP-seq
☒	☐	Flow cytometry
☐	☒	MRI-based neuroimaging

Eukaryotic cell lines

Policy information about cell lines

Cell line source(s)	HEK293
Authentication	Cell lines were not independently authenticated.
Mycoplasma contamination	Cell lines tested negative for mycoplasma contamination.
Commonly misidentified lines (See ICLAC register)	n/a

Animals and other organisms

Policy information about studies involving animals; ARRIVE guidelines recommended for reporting animal research

Laboratory animals	All animals were male Sprague-Dawley rats, 8-10 weeks of age, purchased from Charles River Laboratories.
Wild animals	n/a
Field-collected samples	n/a
Ethics oversight	All procedures were performed in strict compliance with US Federal guidelines, with oversight by the MIT Committee on Animal Care.

Note that full information on the approval of the study protocol must also be provided in the manuscript.

Magnetic resonance imaging

Experimental design

Design type	block design

Design specifications	Each in vivo KCl stimulation experiment consisted of a 5 min pre-stimulation period, followed by 5 min of stimulation and 10 min of rest.
Behavioral performance measures	n/a

Acquisition

Imaging type(s)	functional, structural
Field strength	9.4 T
Sequence & imaging parameters	anatomical imaging: RARE (TE = 8 ms, TR = 300 ms, FA = 160°, RARE factor = 4, number of averages = 20); functional imaging: gradient echo (TE = 5.5 ms, TR = 150 ms, FA = 45°)
Area of acquisition	contrast agent infusion area (striatum)

Diffusion MRI ☐ Used ☒ Not used

Preprocessing

Preprocessing software	ImageJ, MATLAB
Normalization	Data were processed using custom scripts for high/low pass filtering, detrending using a polynomial fit to the pre-stimulus baseline and the last data point, with averaging across multiple experiments. Time course images were binned into 2 minute averages centered around the peak response.
Normalization template	n/a
Noise and artifact removal	n/a
Volume censoring	n/a

Statistical modeling & inference

Model type and settings	Unifactorial (test vs. baseline).
Effect(s) tested	Signal response change in response to stimulation or control treatments assessed.

Specify type of analysis: ☐ Whole brain ☐ ROI-based ☒ Both

Anatomical location(s)	For analysis of dynamic signal changes during stimulation, MRI intensity was integrated over ROIs defined by a 1.2 x 1.2 mm square area adjacent to the cannula tip and centered on the point of peak signal change during stimulation period. Percent signal changes were computed with respect to a baseline defined by the first 5 min of imaging and the last time point. Mean signal change amplitudes reported in Fig. 5e were defined by the average signal change during the final 1 min of infusion. The map of percent signal change in Fig. 5d was generated by averaging percent signal change maps from five animals aligned to their respective cannula tips and plotting the result over-laid on a representative anatomical image.
Statistic type for inference (See Eklund et al. 2016)	n/a
Correction	n/a

Models & analysis

n/a	Involved in the study
☒	☐ Functional and/or effective connectivity
☒	☐ Graph analysis
☒	☐ Multivariate modeling or predictive analysis
